# Molecularly Imprinted Polymer-Based Electrogenerated Chemiluminescence Sensor for Sensitive and Selective Fentanyl Detection

**DOI:** 10.1021/acs.analchem.5c06407

**Published:** 2026-01-07

**Authors:** Arati Biswakarma, Wujian Miao

**Affiliations:** Department of Chemistry and Biochemistry, 5104 The University of Southern Mississippi, Hattiesburg, Mississippi 39406, United States

## Abstract

The escalating prevalence of fentanyl, a highly potent synthetic opioid responsible for rising overdose fatalities and public health crises, underscores the critical need for sensitive and selective detection methodologies. Herein, we report a molecularly imprinted polymer (MIP)-based electrogenerated chemiluminescence (ECL) sensor for ultrasensitive and selective quantification of fentanyl. The sensor was fabricated by electropolymerizing 4-aminobenzoic acid (4-ABA) on a glassy carbon electrode (GCE) in the presence of fentanyl as the template, followed by template elution to yield recognition nanocavities within the MIP film. ECL signal transduction was achieved through the anodic coreactant pathway, wherein fentanyl (as the coreactant) bound within the MIP cavities reacted with solution-phase [Ru­(bpy)_3_]^2+^ (as the ECL emitter) in phosphate buffer (pH 7.5) during anodic scans from 0 to 1.60 V vs Ag/AgCl (3.0 M KCl). Critical fabrication and operational parameters, including electropolymerization cycles, template concentration and molar ratio to the monomer, elution conditions, and rebinding duration, were systematically optimized. Density functional theory and density of states investigations guided elution solvent selection and elucidated favorable fentanyl–polymer interactions, verifying the sensor’s specificity toward fentanyl over five common structurally similar interferents. Under optimized conditions, the MIP-ECL sensor exhibited a limit of detection of ∼1 μM (S/N = 3), equivalent to 3.4 ng of fentanyl using 10 μL solution, with three distinctive linear regions spanning 1.0 to 6.0, 6.0 to 40.0, and 40.0 to 70.0 μM. Additionally, the sensor demonstrated high reproducibility and stability, positioning it as a promising platform for rapid forensic analysis of illicit fentanyl.

## Graphical abstract

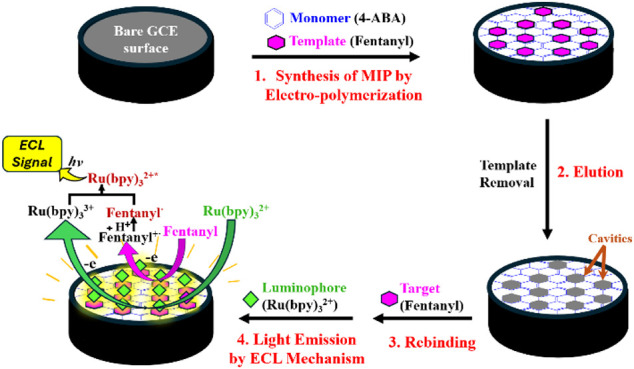

## Introduction

Fentanyl, a synthetic opioid, is 50 and 100 times more potent than heroin and morphine, respectively (Figure 1), and its illicit production poses a significant public health threat.^1^ Grzybowski et al. demonstrated that fentanyl can be synthesized through 166 possible pathways, even when excluding controlled chemicals routes, using chemical artificial intelligence tools like Allchemy and Synthia, increasing the risk of illegal manufacturing.^2^ Illegally produced fentanyl, primarily from overseas, is smuggled into the United States through borders and sold as counterfeit pills or mixed with other street drugs.^2–4^ In 2022, fentanyl contributed to 70% of the total 109,000 drug overdose deaths in the United States.^5^ This crisis underscores the urgent need for sensitive and selective sensors to detect fentanyl at U.S. ports of entry or borders,^6^ clinical settings,^7^ and forensic examinations.^8^

**Figure 1:**
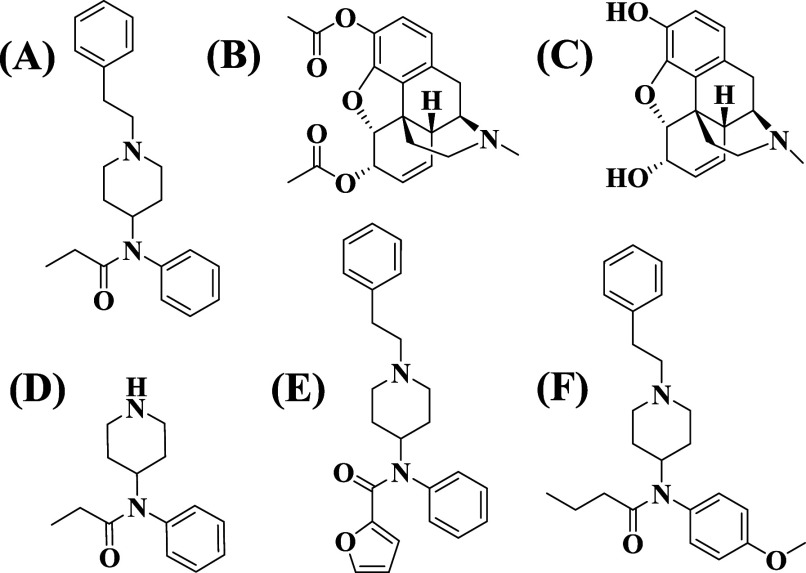
Molecular structures of (A) fentanyl and its potential interferents for detection: (B) heroin, (C) morphine, (D) norfentanyl, (E) furanyl fentanyl, and (F) para-methoxy-butyryl fentanyl.

Several methods have been developed for fentanyl detection, including liquid chromatography/mass spectrometry (LC/MS),^9^ surface-enhanced Raman spectroscopy (SERS),^10^ immunoassays,^11^ thermal desorption direct analysis in real time mass spectrometry (TD-DART-MS), ion mobility spectrometry (IMS),^12^ colorimetric methods,^13^ ultrahigh-performance liquid chromatography with tandem mass spectrometry (UHPLC-MS/MS),^14^ and gas chromatography with mass spectrometry (GC-MS).^15^ Although these techniques are highly sensitive, they require complex instrumentation, skilled personnel, and are costly, nonportable, and susceptible to background signals from interferents.^16,17^ On the other hand, electrochemical probes using techniques such as cyclic voltammetry (CV),^18^ differential pulse voltammetry (DPV),^19^ square wave voltammetry (SWV),^20^ and adsorptive stripping voltammetry (AdSV)^21^ have been established for fentanyl analysis due to their ease of use, low cost, sensitivity, and potential for miniaturization.^22^ However, these approaches often suffer from limited reproducibility, stability, and selectivity due to interferent adsorption and challenges in detecting low concentrations.^23^

Molecularly imprinted polymers (MIPs) offer selective recognition properties.^24^ First reported by Mosbach in 1994 for noncovalent imprinting,^25^ MIP sensors have been used to analyze antibiotics,^26^ harmful chemicals,^27^ biomarkers,^28^ and environmental pollutants.^29^ They provide high specificity, chemical and storage stability, low cost, and versatility for various molecular analytes.^30^

Electrogenerated chemiluminescence (ECL) is an effective analytical technique in which light is emitted by excited state species formed through high energy electron transfer reactions at the electrode surface. Studied since 1964, ECL has been applied across biomedical, medicinal, forensic, and environmental fields to detect, e.g., biological warfare agents, pollutants, and food and water contaminants.^31,32^ ECL offers high sensitivity, cost-effectiveness, low background signals, and ease of control.^33^

This study combines MIP and ECL techniques to develop a MIP-based ECL sensor that leverages their respective advantages for selective and sensitive fentanyl detection at low concentrations and nanogram masses. As illustrated in Scheme 1, 4-aminobenzoic acid (4-ABA) was selected as the monomer and fentanyl as the template. The 4-ABA was electropolymerized with the fentanyl template on a glassy carbon electrode (GCE) surface, followed by template elution to form the MIP sensor. After rebinding the target fentanyl, ECL studies were conducted. The tertiary amine piperidine group of fentanyl oxidizes at the electrode surface at ca. 0.8 V vs Ag/AgCl.^34^ During ECL measurements, the surface-confined fentanyl served as the coreactant, and tris­(2,2′-bipyridyl)­ruthenium­(II) ([Ru­(bpy)_3_]^2+^) in the electrolyte solution acted as the emitter, producing an ECL signal proportional to the amount of the target trapped within the MIP via classic anodic coreactant ECL pathways.^35^ Unlike our recent ECL-MIP report on sensitive and selective detection of the hallucinogenic drug *N,N*-dimethyltryptamine, where the ECL emitter [Ru­(bpy)_3_]^2+^ was embedded within a Nafion layer on electrode,^36^ the present approach places the fabricated GCE|MIP⊂fentanyl electrode in a [Ru­(bpy)_3_]^2+^ solution, simplifying sensor fabrication and enhancing ECL signal sensitivity. Density functional theory (DFT) and density of states (DOS) studies were performed to select a suitable solvent for fentanyl solubility, investigate monomer/polymer-template interactions, and confirm the MIP’s selectivity for fentanyl over potential interferents (Figure 1). A very recent study describing a polydopamine-based MIP electrochemical sensor for fentanyl determination has been published.^37^ However, to our knowledge, MIP-based ECL sensors have not been previously reported for fentanyl analysis. Our sensor meets the requirements for efficient fentanyl detection, as validated by theoretical and experimental studies.

**Scheme 1:**
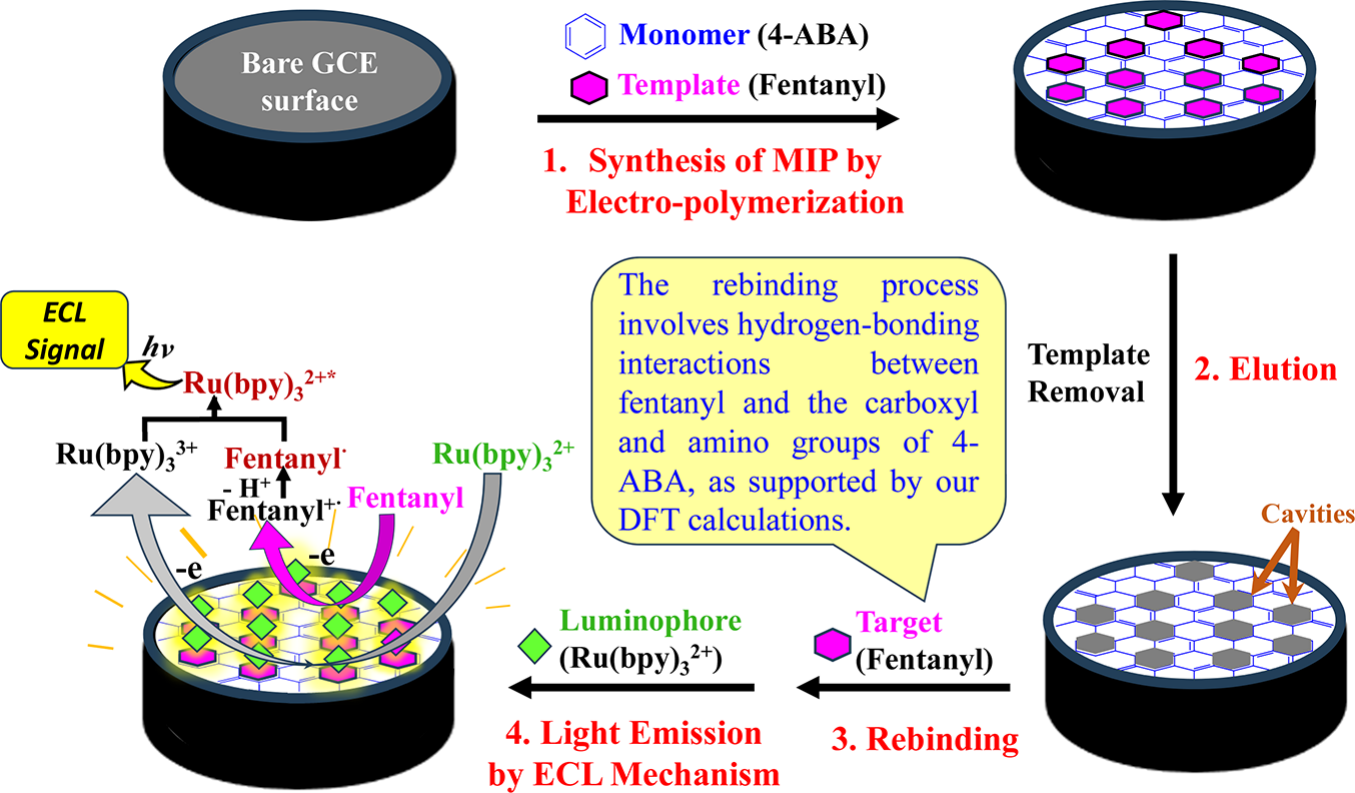
Schematic Representation of the Operational Principle of the MIP Based ECL Sensor

## Experimental Section

### Reagents

All chemicals were used as received, with details provided in the Supporting Information. A 0.10 M phosphate buffer solution (PBS, pH 7.5) was used as the working solution. To eliminate potential redox interference from methanol and MeCN, all standard reference solutions were reconstituted in 0.10 M PBS (pH 7.5) after the initial solvents were slowly evaporated under a nitrogen stream.

### Electrochemical and ECL Studies

Cyclic voltammetry (CV) was conducted with a Model 660A electrochemical workstation (CH Instruments, Austin, TX). ECL signals with CV responses were recorded using a custom-built ECL instrument, as described previously,^33^ with a DC potential of −700 V applied to a Hamamatsu R928 photomultiplier tube. The three-electrode electrochemical cell system consisted of a 3.0 mm diameter glassy carbon electrode (GCE) as the working electrode, an Ag/AgCl (3.0 M KCl) reference electrode, and a Pt mesh counter electrode for the CV and ECL measurements.

### Fabrication of MIP and NIP Films on Bare GCE and Assembly of the MIP Sensor

The bare GCE was polished using 0.3–0.05 μm alumina slurry, washed with water, ultrasonicated in water for several minutes, rinsed again, wiped with Kimwipes, and then allowed to dry in air. For MIP polymerization, an electrolyte solution containing 4.0 mM 4-ABA and 1.0 mM fentanyl in 0.10 M PBS (pH 7.5) was scanned at 50 mV/s from −0.2 to 1.0 V vs Ag/AgCl for 10 cycles using the GCE. The nonimprinted polymer (NIP) was similarly prepared using only 4.0 mM 4-ABA in the same PBS solution.

The fabricated MIP-modified GCE was air-dried, then immersed in 10 mL of MeOH:HAc (9:1 v/v) elution solvent for 10 min to remove residual template, followed by PBS rinsing (swirling several times) and air-drying. For rebinding, 10 μL of fentanyl solution was pipetted onto the MIP surface and incubated for 10 min; excess solution was removed, and the electrode was air-dried. Unbound fentanyl was washed by immersion in 5 mL PBS with several swirls, followed by further drying. The resulting assembly is denoted as GCE|MIP⊂fentanyl, where “|” represents the GCE-MIP interface, and “⊂” indicates fentanyl inclusion within the MIP matrix. ECL measurements were performed by immersing the dried electrode in 3 mL of 0.70 mM [Ru­(bpy)_3_]^2+^ in 0.10 M PBS (pH 7.5). Post-ECL, the electrode was cleaned via immersion in 5 mL deionized water with 10 swirls and air-dried. Sensor regeneration was achieved by repeating the elution procedure.

### Synthesis and Characterization of Fentanyl

Fentanyl was synthesized using a modified one-pot synthesis approach based on Asadi et al.^38^ Briefly, 4-piperidone monohydrochloride was dissolved in dichloroethane, followed by the addition of triethylamine and phenylacetaldehyde, then sodium triacetoxyborohydride and stirred for 48 h under a nitrogen atmosphere. Aniline, glacial acetic acid, and additional sodium triacetoxyborohydride were added, with stirring for 24 h, followed by propionyl chloride for 6 h to yield crude fentanyl. The mixture was diluted with dichloromethane, washed with 4% aqueous NaOH and water, extracted with 2.0 M HCl, and the organic layer was dried and evaporated to yield crude fentanyl hydrochloride (Scheme S1 in Supporting Information). Recrystallization from acetone, followed by treatment with 20% aqueous NaOH and recrystallization from petroleum ether, afforded pure fentanyl as a white powder, characterized by ^1^H NMR (Figure S1 in Supporting Information), FTIR (Figure S2 in Supporting Information), and MS (Figure S3 in Supporting Information). Detailed fentanyl synthesis and characterization are provided in the Supporting Information.

### Safety Note

According to the OSHA Hazard Communication Standard (HCS) safety data sheet (SDS) from Cayman Chemical, fentanyl poses acute toxicity risks and can be fatal if inhaled, ingested, or absorbed through the skin. Proper handling requires personal protection equipment (e.g., impermeable gloves), access to respiratory protection, and adequate workplace ventilation.

## Results and Discussions

### CV and ECL Studies of Fentanyl on Bare GCE

Figure 2A shows the cyclic voltammograms (CVs) of (a) 0.70 mM standard and (b) 0.79 mM synthesized fentanyl in 0.10 M PBS (pH 7.5) within a potential range of 0 to 1.60 V vs Ag/AgCl at a GCE. A larger oxidation current is observed in (b) compared to (a), which can be attributed to the difference in fentanyl concentration, as predicted by the Randles–Sevcik equation.^39^ The redox behavior of the two fentanyl solutions is essentially identical, confirming the successful synthesis of fentanyl in our laboratory. Two irreversible oxidation waves are observed, with peak potentials for the first (Ox1) and the second anodic process (Ox2) at approximately 1.02 and 1.20 V vs Ag/AgCl, respectively. Based on previous studies of the anodic oxidation of nitrogen-containing compounds,^40^ particularly tri-*n*-propylamine (TPrA)^41^ and fentanyl and its derivatives,^19,21,34,42,43^ Scheme 2 illustrates a proposed step-by-step reaction mechanism under the present experimental conditions. With an experimentally reported p*K*_a_ ≈ 8.4,^44^ approximately 89% of fentanyl is estimated to be protonated at pH 7.5. Consequently, predeprotonation of protonated fentanyl (FentH^+^) is a prerequisite step for anodic oxidation. The mechanism of the Ox1 process follows an electrochemical-chemical-electrochemical (ECE) pathway, involving a two-electron, two-proton transfer coupled with dealkylation and subsequent hydrolysis. Specifically, the tertiary amine of fentanyl (Fent) undergoes a one-electron oxidation to form a fentanyl radical cation (Fent^•+^), which subsequently deprotonates to produce a fentanyl free radical (Fent^•^), a key species with strong reducing power required for ECL generation (see later discussion for details). The free radical further oxidizes on the electrode surface, forming an iminium ion intermediate (Fent-iminium) that hydrolyzes to yield norfentanyl and 2-phenylacetaldehyde. Similar to the TPrA oxidation,^41^ the second anodic electron transfer (i.e., the oxidation of Fent^•^ to Fent-iminium) does not generate a separate anodic wave due to the highly negative redox potential of the Fent-iminium/Fent^•^ couple. The Ox2 process begins with the one electron oxidation of norfentanyl (Norfent, a secondary amine) to form a radical cation (NorFent^•+^), which rapidly deprotonates to produce a free radical (NorFent^•^) with a high reducing capacity needed for anodic coreactant ECL. A norfentanyl iminium ion (Norfent-iminium) is subsequently formed via the one-electron oxidation of the free radical, followed by hydrolysis to yield a fentanyl metabolite (Fent-metabolite). The trends and peak potential values shown in Figure 2A are consistent with the anodic oxidation of tertiary amines occurring at less positive potentials than those for secondary amines.^45^ Note that the side product 2-phenylacetaldehyde from the Ox1 process cannot be oxidized within an aqueous electrolyte solution.^46^

**Figure 2:**
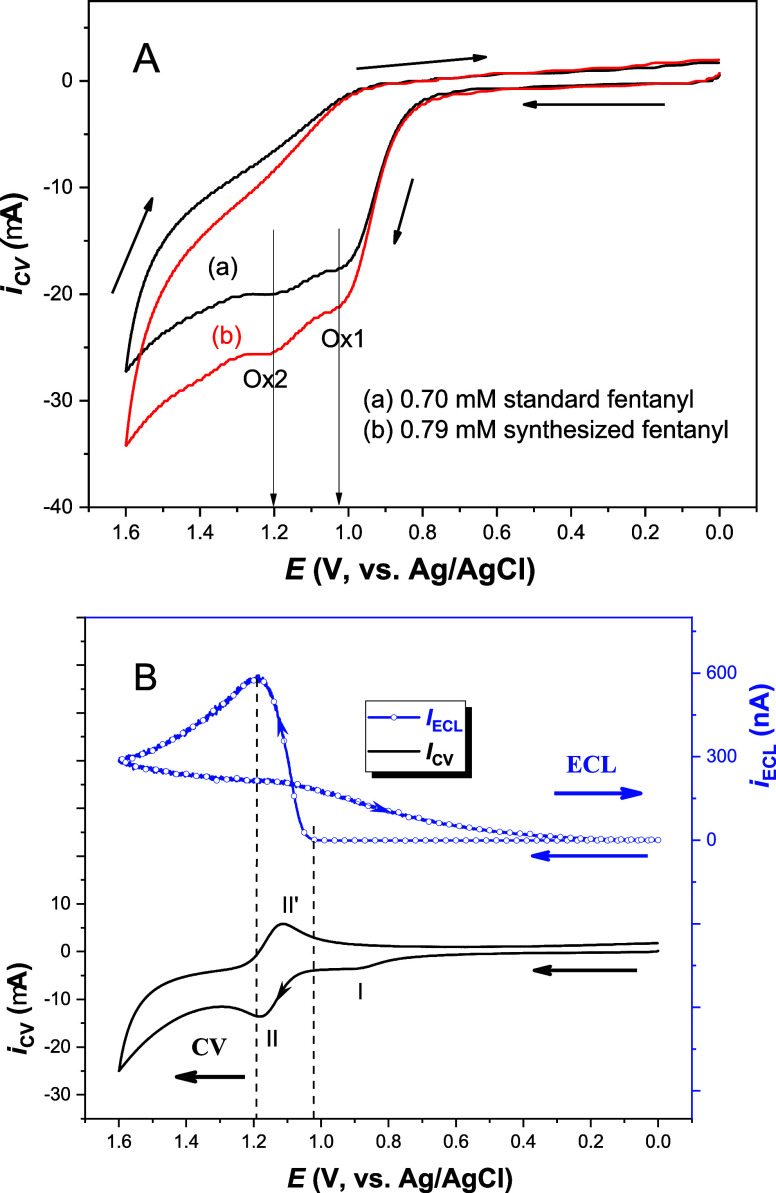
(A) Comparison of CVs between (a) 0.70 mM standard and (b) 0.79 mM synthesized fentanyl in 0.10 M PBS (pH 7.5) at a GCE with a scan rate of 50 mV/s. (B) CV and ECL responses of 50 μM fentanyl with 1.0 mM [Ru­(bpy)_3_]^2+^ in 0.10 M PBS (pH 7.5) at a GCE and a scan rate of 50 mV/s.

**Scheme 2:**
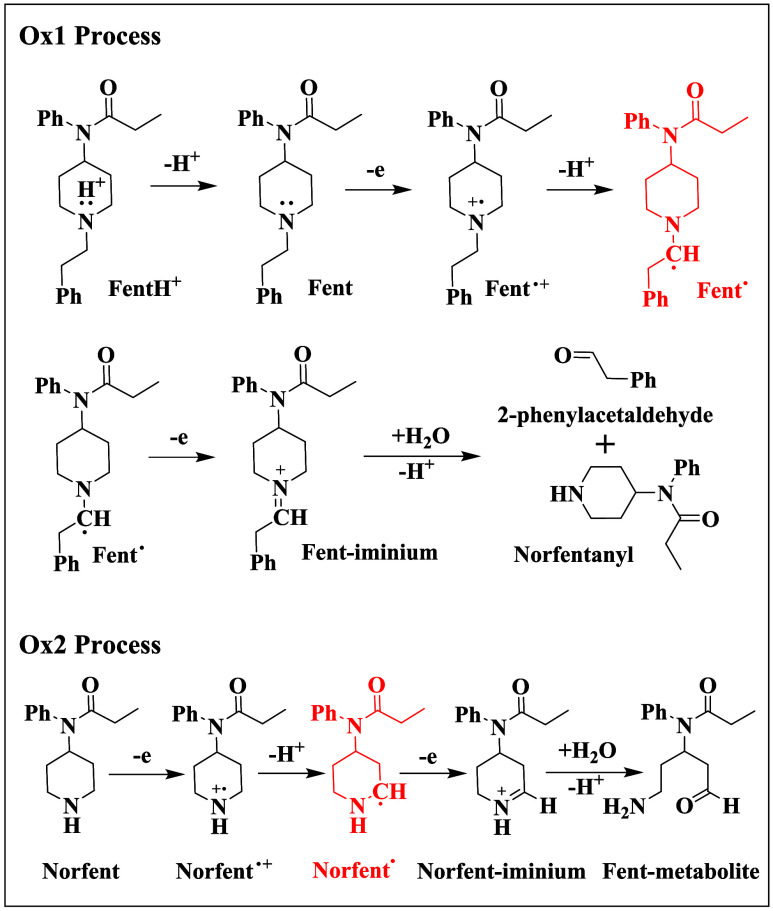
Proposed Anodic Oxidation Mechanism of Fentanyl in 0.10 M PBS at pH 7.5 on a GCE

Figure 2B displays the CV (black curve) and ECL (blue curve) responses of 50 μM fentanyl mixed with 1.0 mM [Ru­(bpy)_3_]^2+^ in 0.10 M PBS (pH 7.5) at a GCE. A small irreversible oxidation wave at ∼0.9 V vs Ag/AgCl (wave I), corresponding to the first anodic oxidation process of fentanyl (i.e., the Ox1 process in Scheme 2), is observed. This is followed by a reversible redox reaction process of [Ru­(bpy)_3_]^2+/3+^ with a half-wave potential of 1.14 V vs Ag/AgCl (wave II/II′). The electrogenerated [Ru­(bpy)_3_]^3+^ can chemically oxidize fentanyl to form Fent^•^, while itself returns to [Ru­(bpy)_3_]^2+^ via a catalytic (EC′) pathway,^47^ which favors ECL formation. Electrochemical oxidation of norfentanyl at potentials beyond ∼1.1 V vs Ag/AgCl is also expected; however, due to its low concentration and overlap of its oxidation potential with that of [Ru­(bpy)_3_]^2+^, no distinct anodic wave is visible in the CV. Regarding the ECL response, light emission is detected only after the oxidation of both fentanyl and [Ru­(bpy)_3_]^2+^. The hysteresis and crossover in ECL intensity during the reverse potential scan are likely attributed to the continued ECL generation associated with norfentanyl oxidation at high positive potentials. Scheme 3 depicts the proposed ECL mechanism for the fentanyl/[Ru­(bpy)_3_]^2+^ system in 0.10 M PBS (pH 7.5) at a GCE. Three pathways (eqs 6, 7, and 10) are proposed for generating the excited state [Ru­(bpy)_3_]^2+^* species. Accordingly, increasing the concentration of [Ru­(bpy)_3_]^2+^ (Figure S4 in Supporting Information) or fentanyl enhances ECL intensity. Note that all electrochemical steps and subsequent chemical reactions responsible for ECL generation, including those involving radical intermediates, occur at the working electrode surface or within its surrounding diffusion layer.

**Scheme 3:**
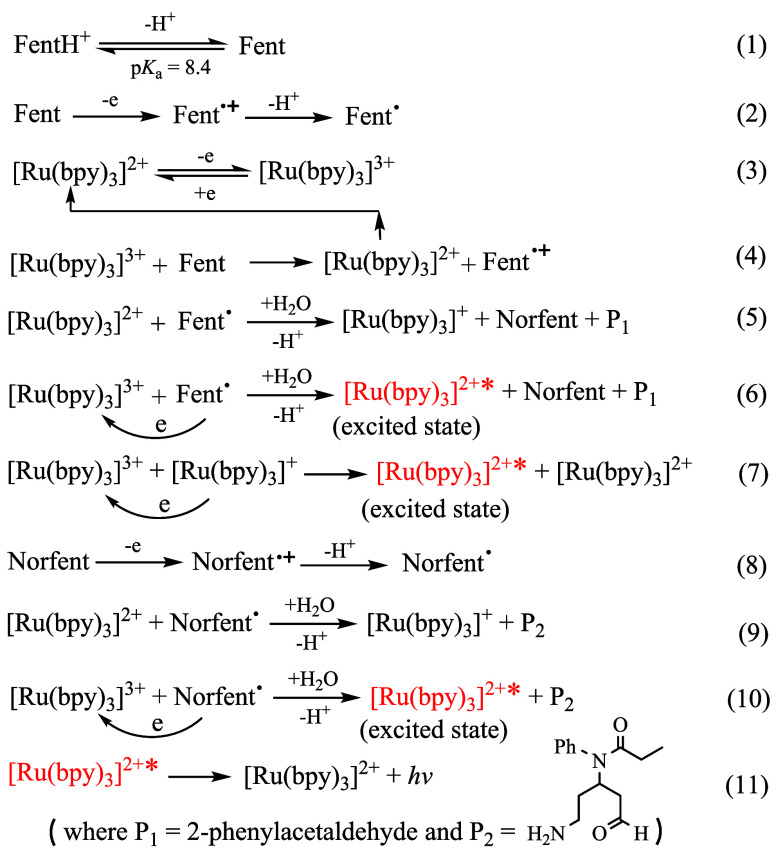
Proposed ECL Mechanism for the Fentanyl/[Ru­(bpy)_3_]^2+^ System in 0.10 M PBS (pH 7.5) at a GCE

### Electropolymerization of MIP and NIP

The MIP and NIP were prepared via electropolymerization of 4-ABA in the presence and absence of template fentanyl, respectively, in 0.10 M PBS (pH 7.5) at a GCE. Similar CV behavior was observed in both cases, as a relatively low concentration of fentanyl (1.0 mM) compared to 4-ABA (4.0 mM) was used, and a maximum anodic potential of 1.0 V vs Ag/AgCl was applied to minimize potential oxidation of fentanyl. Figure 3 shows the CV responses of the first three and the tenth cycles of 4.0 mM 4-ABA mixed with 1.0 mM fentanyl.

**Figure 3:**
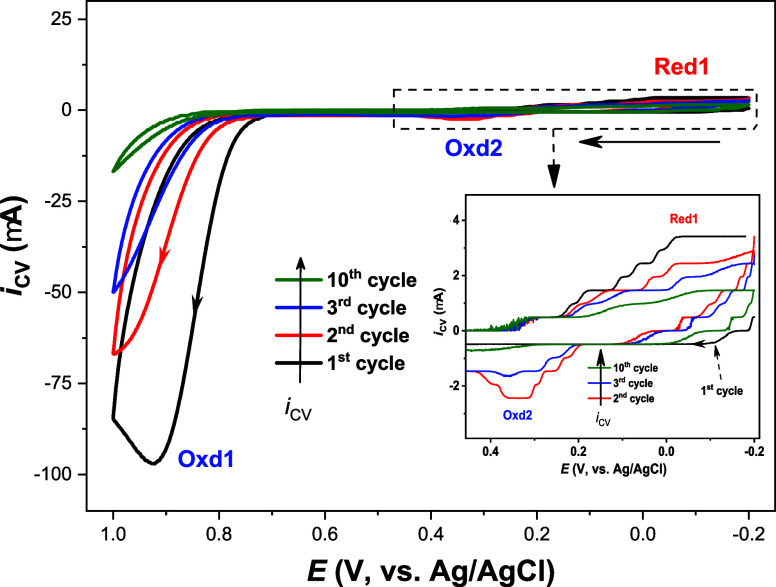
Electropolymerization of 4.0 mM 4-ABA with 1.0 mM fentanyl in 0.10 M PBS (pH 7.5) at a GCE, using a scan rate of 50 mV/s. For clarity, CVs for the 4th to 9th cycles are not shown.

Upon the anodic potential scanning, 4-ABA undergoes irreversible oxidation (Oxd1) with a peak potential of 0.92 V vs Ag/AgCl. This is followed by a small pair of quasi-reversible reduction (Red1 at ∼−0.02 V vs Ag/AgCl) and oxidation (Oxd2 at ∼0.34 V vs Ag/AgCl) waves. Much more distinguishable Red1/Oxd2 waves can be observed for 4-ABA alone at a GCE in 0.10 M PBS (pH 7.4), as we reported previously.^36^ Notably, as the number of CV cycles increases, the redox currents decrease gradually, and the main oxidation waves (Oxd1) shifts to more positive potentials. These observations are consistent with the continuous polymerization of 4-ABA on the electrode surface, which reduces the accessibility of the GCE to redox species and forms an MIP or NIP with higher charge transfer resistance than GCE (see the following section for details).

Studies on the electropolymerization of 4-ABA and its derivatives have been reported under various experimental conditions, including different solvents, pH values, electrodes, and potential ranges.^36,48–53^ Due to the positive mesomeric effect of the amino group in 4-ABA, it directs substituents to the ortho and para positions. Since the para position is already occupied by the carboxylic acid group, substitutions are limited to the ortho positions (C-1 and C-6). Consequently, 4-ABA monomers can interlink through these positions, enabling polymer formation. One proposed pathway for electropolymerization of 4-ABA involves coupling between the resonance forms of the newly electrogenerated cation radicals and neutral molecules,^51^ as we recently suggested in 0.10 M PBS (pH 7.4) at a GCE cycled between −0.20 and 1.00 V vs Ag/AgCl.^36^ An alternative pathway (Scheme 4), potentially more favorable based on recent theoretical and experimental findings,^52,53^ involves electropolymerization initiating between the resonance form of the 4-ABA cation radical (**b**) and another cation radical (**a**), forming a dimer ion (**c**) and dimer cation radical (**d**). After electroreduction at Red1 and reoxidation at Oxd2, the dimer cation radical (**c**) reacts with another resonance form of 4-ABA cation radical (**b**) to generate a trimer cation radical (**f**). Following another redox cycle, **f** couples with **b** to form a tetramer, then a pentamer, and ultimately the polymer.

**Scheme 4:**
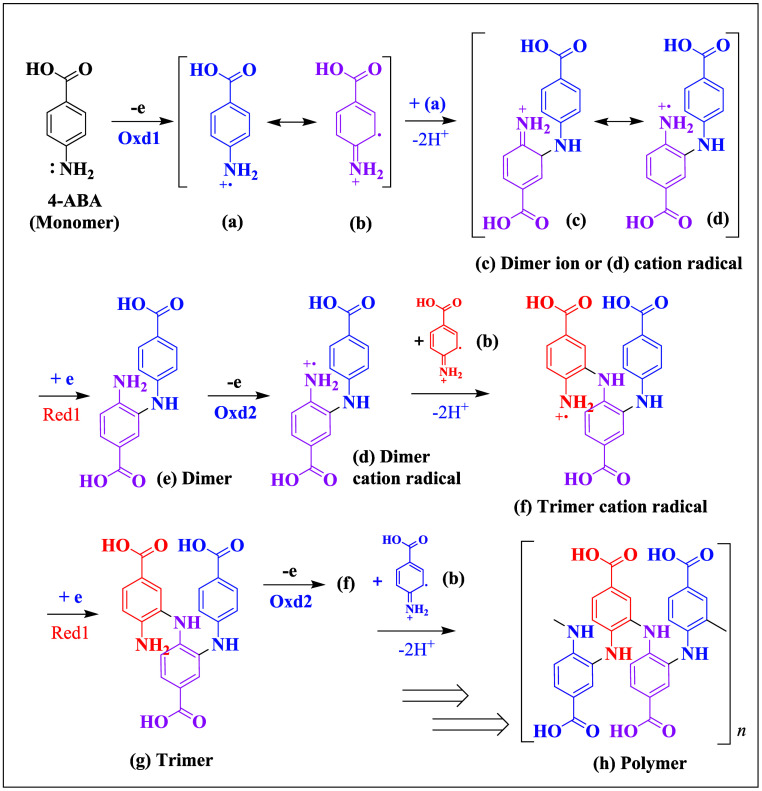
An Alternative Electropolymerization Mechanism of 4-Aminobenzoic Acid (4-ABA) in 0.10 M PBS (pH 7.5) at a GCE with Potentials Cycled between −0.20 and 1.00 V vs Ag/AgCl^a^

a The polymerization process is driven by the consumption of resonance forms of 4-ABA cation radicals. See ref 36 for a proposed electropolymerization mechanism involving neutral molecules in the polymeric chain reactions.

### Electrochemical Characterization of MIP and NIP

The fabricated electrodes were characterized using CV in a neutral aqueous solution containing 4.0 mM K_3_[Fe­(CN)_6_] as the redox standard and 0.10 M KNO_3_ as the supporting electrolyte. As shown in Figure 4A, the well-defined reversible redox behavior on a bare GCE (Figure 4A­(a)) transitions to a fully irreversible process on an MIP electrode (i.e., 4-ABA polymers embedded with saturated fentanyl template, designated as GCE|MIP⊂Fent_satuated_ (Figure 4A­(b)) or an NIP electrode (i.e., 4-ABA polymer film only, Figure 4A­(c)). These observations are attributed to strong repulsive electrostatic interactions between the negatively charged [Fe­(CN)_6_]^3–^ and the negatively charged MIP or NIP film. The vast majority of the −COOH groups in the 4-ABA polymer film are expected to be deprotonated at neutral pH (note: p*K*_a_ for 4-ABA ≈ 2.4 for −COOH),^54^ resulting in a significant decrease in the electron-transfer rate constant. After elution of fentanyl from the MIP, nanocavities are formed on the electrode surface, making [Fe­(CN)_6_]^3–^ more accessible to the GCE substrate (Figure 4A­(d)). Upon rebinding of a low concentration of fentanyl to the eluted MIP, electron-transfer of [Fe­(CN)_6_]^3–^ becomes relatively hindered (Figure 4A­(e)). As predicted, when [Fe­(CN)_6_]^3–^ is replaced with a neutral redox standard, such as ferrocene methanol, the CVs of all fabricated electrodes exhibit responses similar to that of the bare GCE, due to the elimination of repulsive electrostatic interactions (Figure 4B). The slightly larger CV profiles of the fabricated electrodes (Figure 4B­(b)–(e)) compared to the bare GCE (Figure 4B­(a)) are consistent with their surface charge-related double-layer capacitances,^47^ with the NIP electrode (Figure 4B­(c)) exhibiting the most negative charge. By adjusting the electrolyte solution pH from neutral to 3.5, the negative charge density of the fabricated electrodes is expected to be significantly decreased. Consequently, the repulsive electrostatic interactions between [Fe­(CN)_6_]^3–^ and the MIP or NIP films are remarkably reduced, resulting in a shift in their CV behavior from irreversible or quasi-reversible to quasi-reversible or reversible (Figure 4C vs Figure 4A).

**Figure 4:**
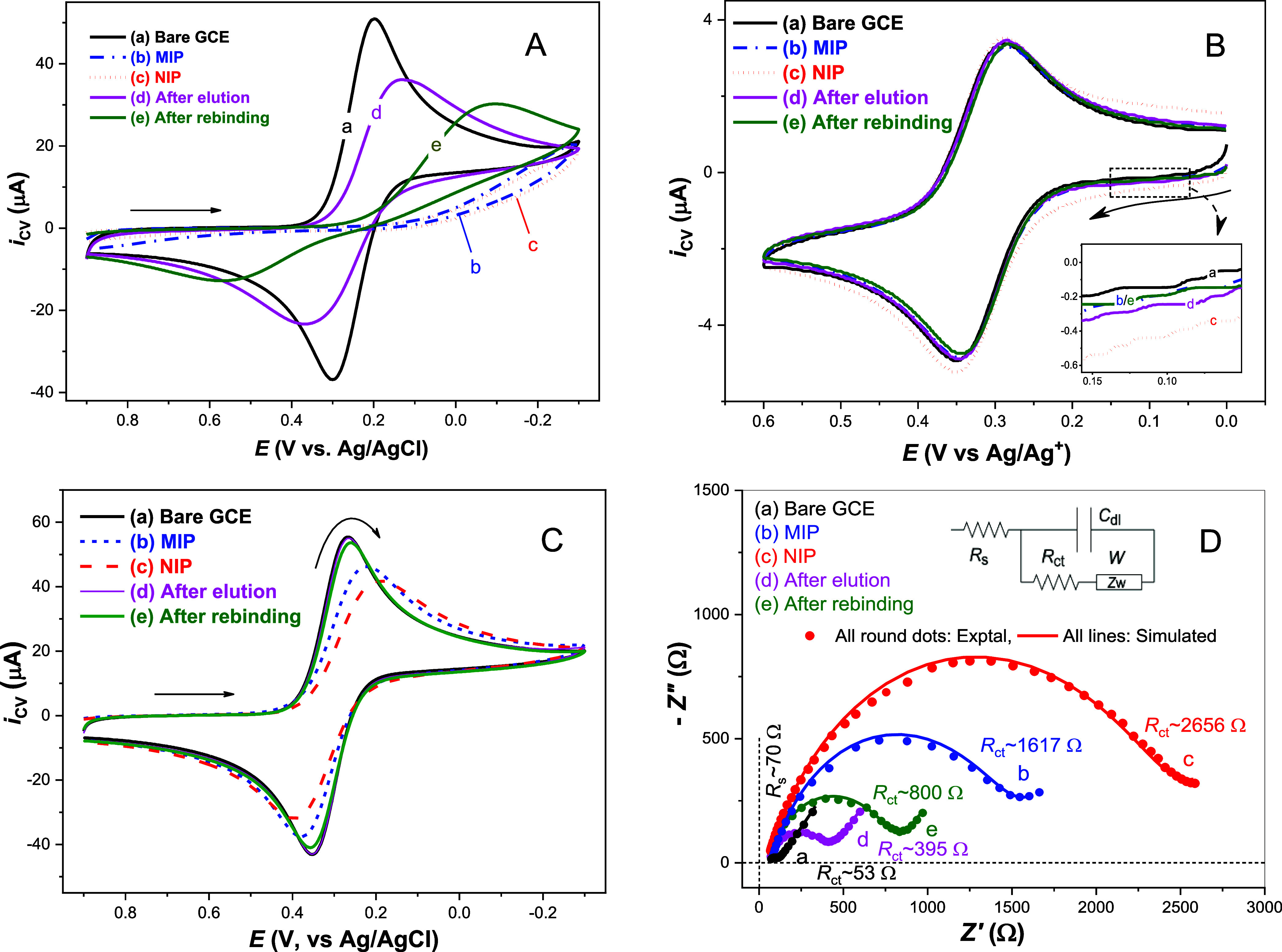
CVs of fabricated electrodes in (A) 4.0 mM K_3_Fe­(CN)_6_ and 0.10 M KNO_3_, (B) 0.50 mM ferrocene methanol and 0.10 M KNO_3_, and (C) 4.0 mM K_3_Fe­(CN)_6_, 0.10 M KNO_3_, and 0.10 M citrate buffer (pH 3.5), with a scan rate of 50 mV/s. The rebinding electrode was prepared using 50 μM fentanyl. (D) Nyquist plots of fabricated electrodes in 5.0 mM K_3_[Fe­(CN)_6_] and 5.0 mM K_4_[Fe­(CN)_6_] mixture with 0.10 M KNO_3_. Insert: Equivalent circuit used to fit EIS data for estimating *R*_s_ and *R*_ct_ values, with *C*_dl_ and *W* representing double-layer capacitance and Warburg impedance, respectively.

Electrochemical impedance spectroscopy (EIS) was used to study the interfacial properties of the fabricated electrode surfaces. EIS measurements were conducted in a solution containing a 5.0 mM K_3_[Fe­(CN)_6_]/5.0 mM K_4_[Fe­(CN)_6_] mixture with 0.10 M KNO_3_ at a formal potential of 0.30 V vs Ag/AgCl, using an alternating voltage of 5 mV. As shown in Figure 4D, all electrodes exhibit nearly identical solution resistance (*R*_s_) of ∼70 Ω, consistent with the use of the same electrolyte solution. However, the charge transfer resistance (*R*_ct_) represented by the semicircle diameter in the Nyquist plot, increases from ∼53 Ω for the bare GCE to 1617 Ω for the MIP electrode and further to ∼2656 Ω for the NIP electrode (Figure 4D­(a)–(c)). After elution, the *R*_ct_ value significantly decreases to ∼359 Ω (Figure 4D­(d)), consistent with the formation of nanocavities on the electrode surface, enhancing diffusion of the [Fe­(CN)_6_]^3–^/[Fe­(CN)_6_]^4–^ redox couple. Upon rebinding of fentanyl to the MIP, the *R*_ct_ value increases to ∼800 Ω (Figure 4D­(e)), confirming successful uptake of the target fentanyl. These results align with the CV data in Figure 4A–C and demonstrate the successful fabrication of the MIP- based ECL sensor.

### Fourier Transform Infrared Spectroscopy (FTIR)

The FTIR spectrum of the solid 4-ABA monomer (Figure S5­(a) in Supporting Information) closely matches literature data,^53,55^ exhibiting characteristic peaks at ∼3456 and ∼3360 cm^–1^ for the primary amino group (−NH_2_), ∼3229 cm^–1^ for the O–H stretch of the carboxylic acid (−COOH), ∼1656 cm^–1^ for the C=O stretch, ∼1278 cm^–1^ for the C–N stretch, and ∼1159 cm^–1^ likely for the C–O in −COOH. The FTIR spectra of the MIP (Figure S5­(c)) and NIP (Figure S5­(d)) films, electrodeposited on a gold-coated silicon wafer (Figure S5­(a)), are nearly identical, indicating that embedded fentanyl molecules in the MIP have a negligible effect on the FTIR spectra. After polymerization, the “free” −NH_2_ groups are transformed into C–NH–C bridges (see Scheme 4 for details), and hydrogen bonding, such as that between the carboxylic acid and the amine groups, is significantly weakened or eliminated due to steric constraints in the polymer matrix. Consequently, the C=O, C–N, and C–O vibrations shift to higher wavenumbers^56^ at ∼1742 cm^–1^ (shifted by 86 cm^–1^), ∼1367 cm^–1^ (shifted by 89 cm^–1^), and ∼1217 cm^–1^ (shifted by 58 cm^–1^), respectively.

### Optimization of MIP-ECL Sensors

#### Concentrations of Fentanyl Template and ECL Emitter [Ru­(bpy)_3_]^2+^

To minimize the contribution of possible fentanyl oxidation to the electropolymerization of 4-ABA in 0.10 M PBS (pH 7.5) (Figure 2A vs Figure 3), while still providing sufficient imprinting sites in the MIP, a fentanyl concentration of 1.0 mM was selected together with a constant 4.0 mM 4-ABA monomer. At concentration above ∼1 mM, fentanyl began to precipitate. Although the ECL intensity of the MIP increased with higher [Ru­(bpy)_3_]^2+^ concentrations (Scheme 3), the blank signal from the NIP also rose, likely due to side reactions associated with the polymer film and the electrolyte at elevated [Ru­(bpy)_3_]^2+^ levels (sub-mM range). Therefore, an optimal concentration of 0.70 mM [Ru­(bpy)_3_]^2+^ was chosen, as this condition produced the maximum ECL difference between the MIP fabricated with 60 μM fentanyl and the NIP.

#### Number of Electropolymerization Cycles

The number of CV cycles determines the thickness of the polymer film on the electrode; a thicker film allows more template molecules to be embedded, thereby increasing the ECL emission. However, excessively thick polymer films reduce the efficiency of interfacial electron-transfer reactions between the entrapped analyte species in the MIP and the solution-phase [Ru­(bpy)_3_]^3+/2+^ (eqs 4–6, and 9–10 in Scheme 3) due to hindered diffusion of reactive species through the film to the [Ru­(bpy)_3_]^3+/2+^ at the MIP-electrolyte interface. Experimentally, as shown in Figure 5A, an MIP prepared with 4.0 mM 4-ABA and 1.0 mM fentanyl in 0.10 M PBS (pH 7.5), using 10 CV cycles between −0.20 and 1.00 V vs Ag/AgCl at 50 mV/s, demonstrates the highest ECL response with 60 μM fentanyl. Therefore, 10 potential cycles with the aforementioned experimental parameters were selected as the optimal condition.

**Figure 5:**
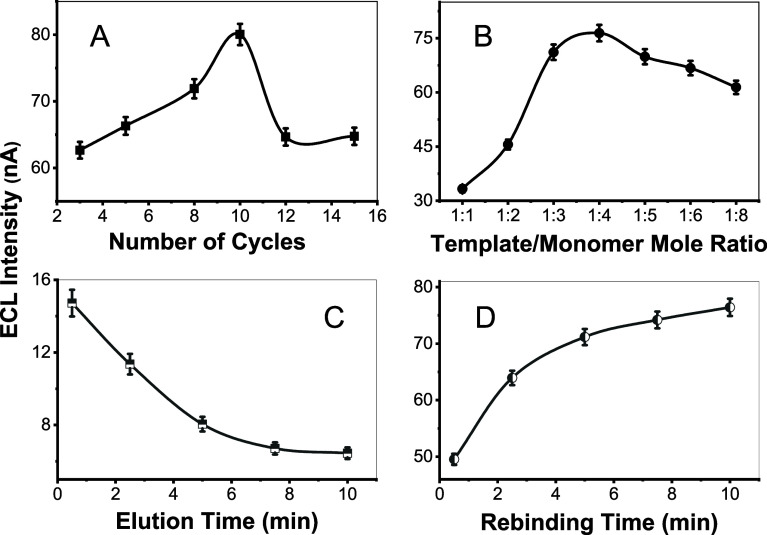
Influences of (A) number of electropolymerization cycles, (B) template-to-monomer ratio, (C) elution time using 9:1 (v/v) MeOH:HAc elution solvent to remove fentanyl from the MIP matrix, and (D) rebinding time of fentanyl target to eluted MIP matrix on the ECL intensities. All ECL signals were measured with MIP-ECL sensors initially prepared from 60 μM fentanyl.

#### Template-to-Monomer Ratio

The molar ratio of template (fentanyl) to monomer (4-ABA) in the preparation of the MIP film significantly influences the polymer’s structure, binding site formation, selectivity, stability, and ECL performance. A higher monomer concentration, such as the 1:4 template-to-monomer ratio (1.0 mM fentanyl to 4.0 mM 4-ABA, Figure 5B) ensures sufficient 4-ABA units to form a stable (4-ABA)*_n_* polymer matrix around fentanyl, creating well-defined binding cavities through interactions like hydrogen bonding. However, an excessively high monomer concentration (e.g., template-to-monomer ratio > 1:8) may lead to overpolymerization, reducing porosity and hindering template removal or analyte rebinding. Figure 5B shows the ECL intensity as a function of the template to monomer molar ratio, with an optimal 1:4 ratio demonstrating maximum performance.

#### Elution Medium and Time

Various solvents (e.g., methanol, acetone, acetonitrile, and phosphate buffer, with or without added acetic acid or sulfuric acid, 5.0 mL each), agitation methods (e.g., dipping, swirling, ultrasonication, and magnetic stirring), and time periods (0.5 to 45 min) were examined for effective elution of fentanyl from the MIP matrix (Table S1 in Supporting Information). Ultimately, a solution mixture of 9:1 (v/v) methanol (MeOH) to pure acetic acid (HAc) was selected as the elution solvent,^57^ which exhibits a minimal and stable ECL signal after the GCE|MIP⊂Fent electrode was dipped in the medium for 10 min (Figure 5C).

Results from density functional theory (DFT) and density of states (DOS) calculations (Section 4 under Results and Discussions in Supporting Information) support the experimental selection of the MeOH-based solvent. Among all calculated solvents, MeOH and ethanol exhibit the lowest positive solvation free energies (Figure S6A). Compared to ethanol, MeOH has a relatively smaller dipole moment and lower polarizability (Figure S6B and C), coupled with a higher HOMO–LUMO energy gap for fentanyl in MeOH (Figure S7).

Although HAc alone is not an efficient elution solvent due to its higher solvation energy (Figure S6A), its addition to MeOH enhances elution by creating an acidic medium that disrupts the hydrogen bonding between fentanyl (template) and the 4-ABA matrix through competitive binding.^36,58^ Consequently, the 9:1 (v/v) MeOH:HAc mixture emerges as an effective fentanyl elution solvent, effectively solubilizing the template while preserving MIP cavity integrity.

#### Rebinding Time

This refers to the duration required for the fentanyl target to rebind to the electropolymerized film within the MIP cavity (formed after fentanyl template removal). ECL signals were recorded starting at a rebinding time of 30 s, but these exhibit weak intensities (Figure 5D). Increasing the rebinding time enhances the ECL responses. No significant change in ECL intensities was observed between 7.5 and 10 min. Consequently, a rebinding time of 10 min was selected as the optimal condition.

#### Calibration Curve and Limit of Detection

Figure 6 shows the calibration curve for ECL detection of fentanyl across a concentration range of 1.0 to 100.0 μM. The curve exhibits three distinct linear regions (*a*, *b*, and *c*) followed by a leveling-off region (*d*). Least-squares linear fitting equations and corresponding *R* ^2^ values for regions *a*–*c* are provided adjacent to their respective lines. This atypical signal-concentration relationship aligns with the three-dimensional, multilayer nanocavity structure of the MIP matrix. It reflects a multistep binding mechanism for fentanyl to the MIP: initial monolayer interactions at low concentrations (region *a*), progressing to multilayer interactions at higher concentrations (regions *b* and *c*), and culminating in saturation (region *d*). Similar behavior has been reported previously for nitrogen adsorption isotherms in microporous or mesoporous network polymers,^59,60^ as well as for *N*,*N*-dimethyltryptamine binding to electropolymerized 4-ABA MIPs.^36^ Under the present experimental conditions, the fabricated MIP-ECL sensor has an estimated limit of detection of ∼1 μM, corresponding to an absolute fentanyl (336.47 g/mol) mass of 3.4 ng in a 10 μL sample solution. Table S2 in the Supporting Information compares limits of detection for fentanyl using various approaches, including electrochemical, visual calorimetry, surface-enhanced Raman spectroscopy, lateral flow chromatographic immunoassay, and nano-CL-EI-MS, with a widespread of detection limits from 0.004 μM using nano-CL-EI-MS^61^ to 27.8 μM using graphene modified microneedle square wave voltammetry.^62^ Although nano-CL-EI-MS provides exceptional sensitivity, it relies on large, high-cost, nonportable instrumentation and extensive sample handling. In contrast, the present MIP-ECL sensor operates with inexpensive materials, minimal sample preparation, and compact detection hardware suitable for decentralized analysis. Relative to less selective electrochemical platforms, the MIP layer offers enhanced molecular recognition and maintains performance even in the presence of potential interferents such as norfentanyl and urine matrix components (see Selectivity section for details). These considerations underscore that, despite a higher LOD than MS-based methods, the MIP-ECL sensor offers practical advantages in portability, cost, operational simplicity, and selectivity that complement existing analytical approaches.

**Figure 6:**
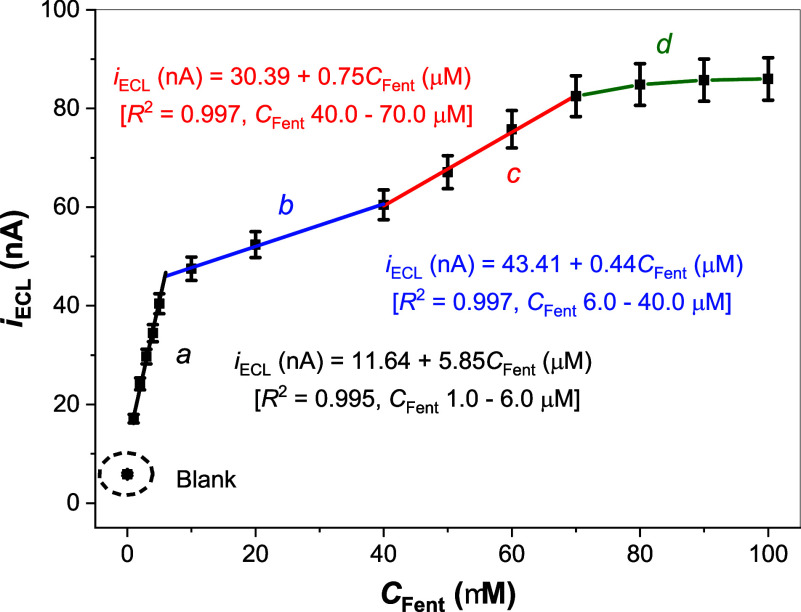
ECL intensity as a function of fentanyl concentration, using 10 μL of standard fentanyl solutions cast on the electrode placed in 0.70 mM [Ru­(bpy)_3_]^2+^-0.10 M PBS (pH 7.5). The calibration curve exhibits three linear regions (*a*–*c*) at low concentration ranges and a nonlinear region (*d*) at higher concentrations, where the MIP film approaches saturation.

#### Reproducibility and Stability

Figure 7 shows the reproducibility of (A) four fabricated MIP electrodes and (B) six replicate measurements with the same MIP electrode, with a relative standard derivation (RSD%) of 2.6% and 5.7%, respectively. The MIP electrode stability was examined by comparing changes in ECL signals regenerated from a 20.0 μM fentanyl solution after the eluted electrode was stored dry in the dark for a certain period. Relative to the ECL intensity from the freshly fabricated electrode, an approximately 13.5% loss in light emission was recorded for a storage of 68 days. A previous ECL detection of fentanyl citrate using a glassy carbon microsphere paste electrode reported a 20% loss in ECL response after 15 days of storage.^63^ These results demonstrate that the present MIP-ECL sensor possesses high reproducibility and stability, and can be reused multiple times consecutively without fouling.

**Figure 7:**
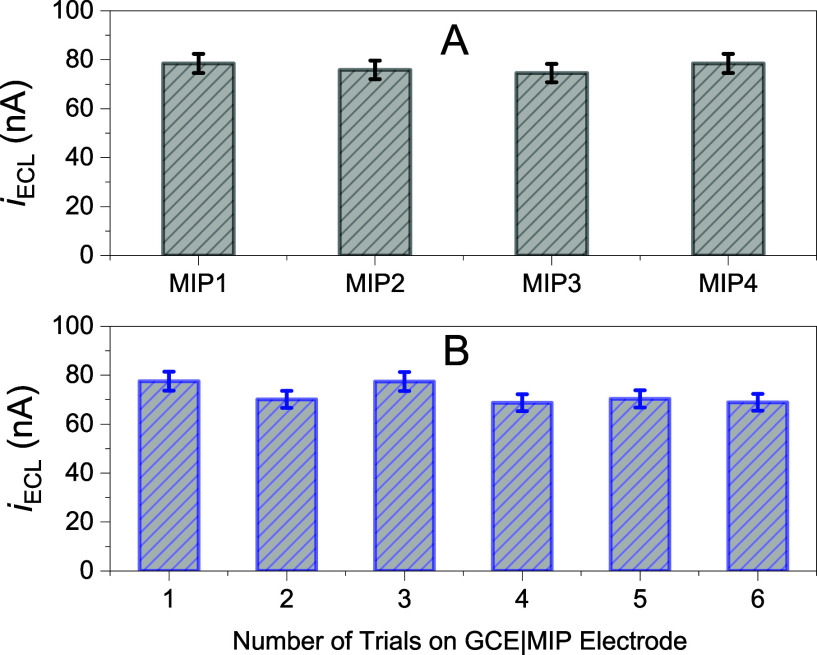
Reproducibility tests of (A) four fabricated MIP electrodes, and (B) six replicate measurements with the same MIP electrode, using 60.0 μM standard fentanyl solutions.

#### Selectivity

ECL responses were measured from MIP electrodes using 20.0 μM fentanyl in the absence and presence of five structurally similar interferents, each at 200.0 μM (see Figure 1 for their names and structures). No notable changes in ECL intensity are observed (Figure 8A) among all tested solution systems, including a mixture containing fentanyl and all five interferents at concentrations 10-times that of fentanyl. The RSD% of the seven trials in the figure is evaluated to be 2.4%, and none of the measurements obtained in the presence of interferents differ significantly from that of fentanyl only at *p* < 0.05, indicating good selectivity of the fabricated MIP-ECL sensor for fentanyl detection.

**Figure 8:**
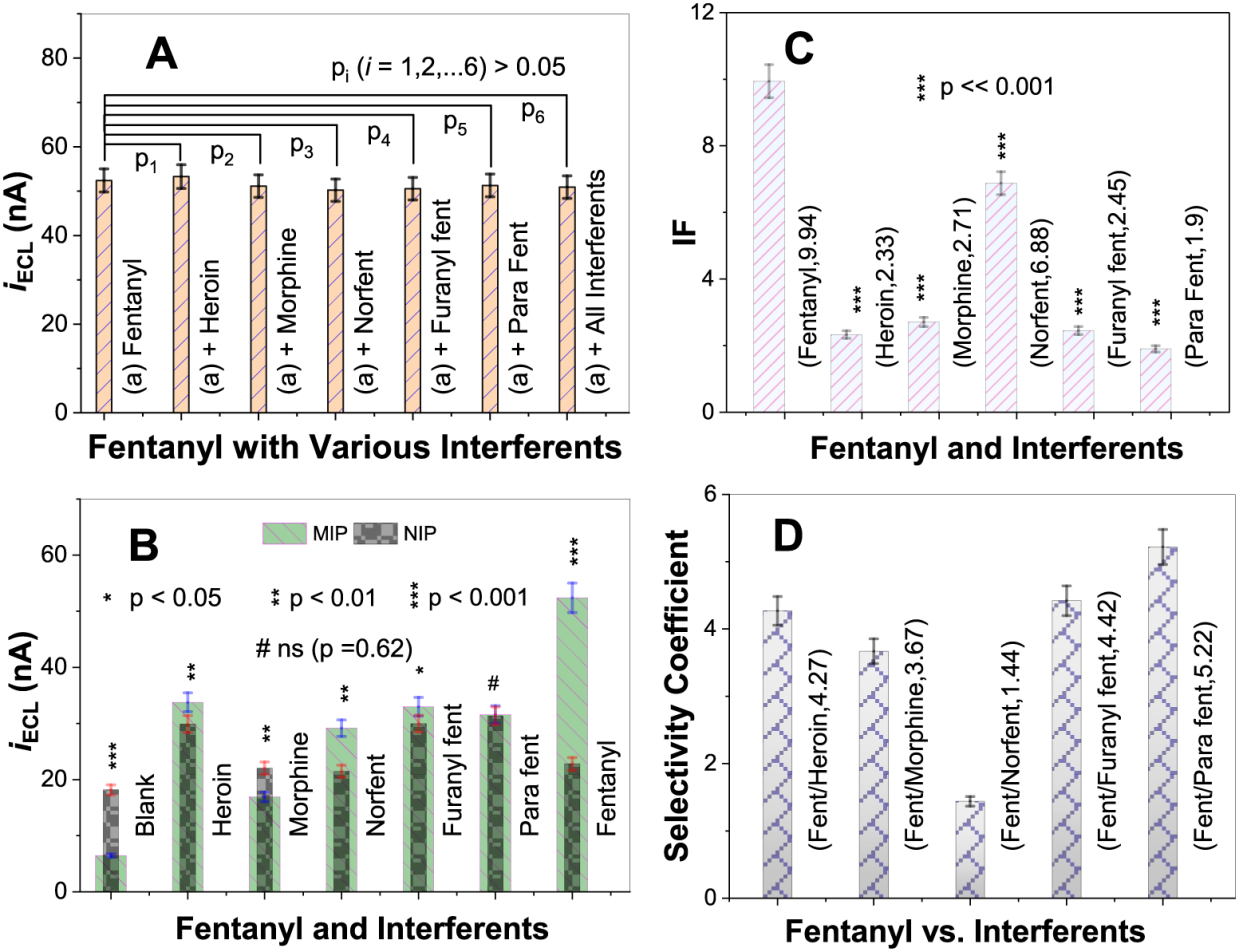
(A) Selectivity tests of the MIP sensor for fentanyl (20.0 μM) in the absence and presence of five structurally similar interferents [heroin (*p*_1_ = 0.294), morphine (*p*_2_ = 0.169), norfentanyl (*p*_3_ = 0.0655), furanyl fentanyl (*p*_4_ = 0.0886), and para-methoxy-butyryl fentanyl (*p*_5_ = 0.199); each at 200.0 μM], as well as in a mixture containing all of the above (*p*_6_ = 0.129). (B) Comparison of ECL responses between MIP and NIP electrodes for the blank, five interferents (each at 200.0 μM), and fentanyl (20.0 μM). (C) Calculated IF values of the MIP sensor for fentanyl and the five interferents. (D) Selectivity coefficients (α) of fentanyl relative to the five interferents. All experiments were conducted in 0.10 M PBS (pH 7.5) containing 0.70 mM [Ru­(bpy)_3_]^2+^.

Figure 8B illustrates the comparison of ECL responses between MIP and NIP electrodes for the blank, five interferents at 200.0 μM, and 20.0 μM fentanyl, respectively. These data are used to calculate the imprinting factor (IF, eq 1)^36^ and the selectivity coefficient (α, eq 2),^64^ two key parameters for assessing the selectivity of MIP sensors, with a high IF or α value indicating high selectivity.

12 $$\mathrm{IF}=\frac{\Delta i_{\mathrm{ECL}}^{\mathrm{MIP}}}{\Delta i_{\mathrm{ECL}}^{\mathrm{NIP}}}=\frac{{\left(i_{\mathrm{ECL}}^{\mathrm{Sample}}-i_{\mathrm{ECL}}^{\mathrm{Blank}}\right)}_{\mathrm{MIP}}}{{\left(i_{\mathrm{ECL}}^{\mathrm{Sample}}-i_{\mathrm{ECL}}^{\mathrm{Blank}}\right)}_{\mathrm{NIP}}}$$

13 $$\alpha =\frac{{\mathrm{IF}}_{\mathrm{Fentanyl}}}{{\mathrm{IF}}_{\mathrm{Interferent}}}$$

where $i_{\mathrm{ECL}}^{\mathrm{Sample}}$ is the ECL intensity measured from a sample containing fentanyl (20.0 μM) or an interferent (200.0 μM), and $i_{\mathrm{ECL}}^{\mathrm{Blank}}$ is the ECL intensity of blank (i.e., no fentanyl or interferent). As expected, fentanyl shows the largest IF value of 9.94, followed by norfentanyl (IF = 6.88), while four other interferents exhibit much smaller IF values between 1.90 and 2.71 (Figure 8C). These IF values reveal that the fabricated MIP electrode is highly selective for fentanyl compared to the interferents heroin, morphine, furanyl fentanyl, and *para*-methoxy-butyryl fentanyl. The relatively lower selectivity for norfentanyl is likely attributed to the fact that, compared to fentanyl, norfentanyl has a smaller molecular structure but similar functional groups, allowing some norfentanyl molecules to bind to the nanocavities designed for fentanyl-specific recognition. Consistently, the high selectivity coefficients (α) of fentanyl relative to all five interferents (α ≈ 4–5, except α = 1.44 for norfentanyl; Figure 8D) indicate that the fabricated MIP nanocavities exhibit high structural and chemical complementarity to the target fentanyl molecule. To illustrate the excellent selectivity of the present fentanyl sensor (IF = 9.94), Table S3 in the Supporting Information compares IF values from some previously reported MIP-based studies, which range from 2.30 to 6.60.

The potential matrix effect on the sensor’s performance was also examined. Specially, urine samples were collected from three volunteers, diluted to 10% (v/v) in 0.10 M PBS (pH 7.5), and spiked with 20.0 μM fentanyl and 200.0 μM norfentanyl. ECL measurements were performed in triplicate for each urine sample. The recovery of fentanyl was calculated as the ratio of the ECL signal obtained from the spiked urine sample to that obtained from 20.0 μM fentanyl in PBS alone. The measured recoveries were 101.71%, 97.14%, and 102.85%, yielding an average of 99.84%. These results indicate that the urine matrix does not adversely affect the performance of the MIP-ECL sensor.

DFT calculations were used to evaluate the MIP’s selectivity based on the interactions (i.e., hydrogen bonding) of three 4-ABA trimers with fentanyl (Figure S8 in Supporting Information) and the interferents in gas and aqueous phases. This approach was selected because the fentanyl/3×(4-ABA)_3_ complex in water exhibited the most negative binding energy and the highest dipole moment among all fentanyl/3×(4-ABA)*_n_* (*n* = 1–3) complexes in both gas and aqueous phases (Figure S9 in Supporting Information), indicating its relatively higher stability. The differences in interactions between fentanyl (and the interferents) and the 3×(4-ABA)_3_ polymer, particularly for fentanyl, followed a consistent trend between gas and aqueous phases: chemical hardness decreased, while chemical softness increased, reflecting a greater propensity for electrophilic or nucleophilic interactions (in this case, hydrogen bonding) upon transitioning from the gas to the aqueous phase^65^ (Figure S10 in Supporting Information). Finally, theoretical selectivity studies for the MIP examined several variables, as detailed in Section 7.2 of the Supporting Information. The binding energy for the interferent heroin indicated unfavorable and nonspontaneous interactions, precluding further investigation. DFT and DOS analyses revealed that fentanyl exhibited nearly the highest chemical hardness (Figure S10A), the lowest chemical softness (Figure S10B), the second most negative binding energy (Figure S11A), the highest dipole moment (Figure S11B), nearly the highest ionization potential (Figure S12A), the most negative chemical potential (Figure S12B), nearly the lowest electrophilicity index (Figure S12C), and nearly the highest HOMO–LUMO gap (Figure S13). Collectively, these factors indicate that the fentanyl/3×(4-ABA)_3_ complex in the aqueous phase is the most stable relative to all interferents. Although furanyl fentanyl displayed the highest binding energy in the aqueous phase (attributable to strong hydrogen bonding between the furan ring oxygen and the trimer, surpassing the weaker nitrogen–trimer interaction of fentanyl; Figure S11A), its overall complex stability, with consideration of other parameters, was lower than that of fentanyl. Similarly, while norfentanyl exhibited the highest HOMO–LUMO gap in the aqueous phase (Figure S13), its binding energy was the lowest among the interferents. A diagrammatic representation of fentanyl interactions (H-bonding) with three 4-ABA trimers in the aqueous phase is shown in Figure S14 in Supporting Information. These findings collectively demonstrate high selectivity of the MIP polymer toward fentanyl over all interferents. This theoretical evidence aligns with prior experimental results, which showed the highest imprinting factor and selectivity coefficient for fentanyl relative to all interferents.

## Conclusion

The fabricated MIP-based ECL sensor demonstrates exceptional sensitivity (with a minimum mass detection of 3.4 ng) and selectivity for fentanyl detection. The sensor was assembled on a GCE employing 4-ABA as the functional monomer and fentanyl as the template molecule. Following electropolymerization, template extraction was conducted with an optimized eluent, thereby generating complementary nanocavities within the MIP matrix to enable selective rebinding of the target analyte. ECL signal transduction was realized via the anodic coreactant ECL mechanism involving [Ru­(bpy)_3_]^2+^ as the luminophore in solution and surface-adsorbed fentanyl as the coreactant. Critical operational parameters, including the number of electropolymerization cycle, template-to-monomer molar ratio, elution medium and duration, and rebinding incubation time, were optimized to maximize sensor performance. Comparative analysis of ECL responses in the presence of fentanyl versus highly concentrated common interferents affirmed the sensor’s high selectivity, along with good reproducibility and stability, as quantified by elevated imprinting factor (IF) and selectivity coefficient (α) values. Complementarily, DFT and DOS computations validated this selectivity theoretically, revealing that fentanyl–MIP interactions in the aqueous phase yield the most thermodynamically stable complex relative to interferents. Overall, this MIP-based ECL sensor represents a reliable, reproducible, and highly selective analytical platform for the identification of controlled substances, such as fentanyl.

## Supplementary Material



## Notes

Reagents; fentanyl synthesis and characterization; ECL studies on bare GCE; FTIR spectra of 4-ABA monomer and polymers on Au/Si; elution of fentanyl from MIP films using various solvents, methods, and time periods; DFT studies for selection of protic and aprotic polar solvents for fentanyl template elution; density of states (DOS) and HOMO–LUMO energy gap studies; comparison of LOD; comparison of the imprinting factor (IF) of MIP sensors; DFT studies for investigating interactions between fentanyl and its interferents and polymers in MIP systems and the selectivity of MIP toward target (PDF)

## References

[1] Poklis A. (1995). Fentanyl:
A Review for Clinical and Analytical Toxicologists. J. Toxicol. Clin. Toxicol.

[2] Mikulak-Klucznik B., Klucznik T., Beker W., Moskal M., Grzybowski B. A. (2024). Catalyst:
Curtailing the Scalable Supply of Fentanyl by Using Chemical AI. Chem.

[3] Walters K. (2018). Fentanyl Trafficking
Trends in the United States. J. Fed. Law Pract..

[4] O’Donnell J., Tanz L. J., Miller K. D., Dinwiddie A. T., Wolff J., Mital S., Obiekwe R., Mattson C. L. (2023). Drug Overdose
Deaths with Evidence of Counterfeit Pill Use — United States,
July 2019–December 2021. MMWR Morb. Mortal.
Wkly. Rep.

[5] Tanz L. J., Gladden R. M., Dinwiddie A. T., Miller K. D., Broz D., Spector E., O’Donnell J. (2024). Routes of
Drug Use Among Drug Overdose
Deaths — United States, 2020–2022. MMWR Morb. Mortal. Wkly. Rep.

[6] Finklea, K. M. Illicit Drug Smuggling Between Ports of Entry and Border Barriers; Congressional Research Service, 2020.

[7] Delaney S. R., Konforte D., Stefan C., Palaty J., Sun D., McDonald K., Thompson H., Werb D., Beriault D. R. (2023). Drug Checking
Services as a Surveillance Tool for Cinical Laboratories: Examining
Trends in the Unregulated Fentanyl Supply. Clin.
Biochem.

[8] Skulska A., Kała M., Parczewski A. (2004). Fentanyl and Its Analogues in the
Forensic Laboratory: Medical and Analytical Problems. Probl. Forensic Sci.

[9] Rab E., Flanagan R. J., Hudson S. (2019). Detection of Fentanyl and Fentanyl
Analogues in Biological Samples Using Liquid Chromatography–High
Resolution Mass Spectrometry. Forensic Sci.
Int.

[10] Haddad A., Comanescu M. A., Green O., Kubic T. A., Lombardi J. R. (2018). Detection
and Quantitation of Trace Fentanyl in Heroin by Surface-Enhanced Raman
Spectroscopy. Anal. Chem.

[11] Schackmuth M., Kerrigan S. (2019). Immunoassay-Based Detection
of Fentanyl Analogs in
Forensic Toxicology. Forensic Toxicol.

[12] Sisco E., Verkouteren J., Staymates J., Lawrence J. (2017). Rapid Detection of
Fentanyl, Fentanyl Analogues, and Opioids for On-Site or Laboratory
based Drug Seizure Screening using Thermal Desorption DART-MS and
Ion Mobility Spectrometry. Forensic Chem.

[13] Mora A. C., Vara M., Reust P., Code A., Oliver P., Mace C. R. (2024). Colorimetric Detection
of Fentanyl Powder on Surfaces
Using a Supramolecular Displacement Assay. ACS
Sens.

[14] Salomone A., Palamar J. J., Bigiarini R., Gerace E., Di Corcia D., Vincenti M. (2019). Detection of Fentanyl Analogs and Synthetic Opioids
in Real Hair Samples. J. Anal. Toxicol.

[15] Leary P. E., Kizzire K. L., Chan
Chao R., Niedziejko M., Martineau N., Kammrath B. W. (2023). Evaluation of Portable
Gas Chromatography–Mass
Spectrometry (GC–MS) for the Analysis of Fentanyl, Fentanyl
Analogs, and Other Synthetic Opioids. J. Forensic
Sci.

[16] Krauss S. T., Ross D., Forbes T. P. (2019). Separation and Detection of Trace
Fentanyl from Complex Mixtures Using Gradient Elution Moving Boundary
Electrophoresis. Anal. Chem.

[17] Goodchild S. A., Hubble L. J., Mishra R. K., Li Z., Goud K. Y., Barfidokht A., Shah R., Bagot K. S., McIntosh A. J. S., Wang J. (2019). Ionic Liquid-Modified Disposable
Electrochemical Sensor
Strip for Analysis of Fentanyl. Anal. Chem.

[18] Jun D., Sammis G., Rezazadeh-Azar P., Ginoux E., Bizzotto D. (2022). Development
of a Graphene-Oxide-Deposited Carbon Electrode for the Rapid and Low-Level
Detection of Fentanyl and Derivatives. Anal.
Chem.

[19] Wester N., Mynttinen E., Etula J., Lilius T., Kalso E., Mikladal B. F., Zhang Q., Jiang H., Sainio S., Nordlund D., Kauppinen E. I., Laurila T., Koskinen J. (2020). Single-Walled
Carbon Nanotube Network Electrodes for the Detection of Fentanyl Citrate. ACS Appl. Nano Mater.

[20] Canoura J., Liu Y., Alkhamis O., Xiao Y. (2023). Aptamer-Based Fentanyl Detection
in Biological Fluids. Anal. Chem.

[21] Ott C. E., Cunha-Silva H., Kuberski S. L., Cox J. A., Arcos-Martínez M. J., Arroyo-Mora L. E. (2020). Electrochemical Detection of Fentanyl with Screen-Printed
Carbon Electrodes using Square-Wave Adsorptive Stripping Voltammetry
for Forensic Applications. J. Electroanal. Chem.

[22] Xue R., Liu Y.-S., Huang S.-L., Yang G.-Y. (2023). Recent Progress
of Covalent Organic Frameworks Applied in Electrochemical Sensors. ACS Sens.

[23] Ferrag C., Kerman K. (2020). Grand Challenges in Nanomaterial-Based
Electrochemical
Sensors. Front. Sens.

[24] Lusina A., Cegłowski M. (2022). Molecularly Imprinted Polymers as State-of-the-Art
Drug Carriers in Hydrogel Transdermal Drug Delivery Applications. Polymers.

[25] Mosbach K. (1994). Molecular
Imprinting. Trends Biochem. Sci.

[26] Hasaneen N., Akhtarian S., Pulicharla R., Brar S. K., Rezai P. (2024). Surface Molecularly
Imprinted Polymer-Based Sensors for Antibiotic Detection. TrAC, Trends Anal. Chem..

[27] Farooq S., Wu H., Nie J., Ahmad S., Muhammad I., Zeeshan M., Khan R., Asim M. (2022). Application
Advancement and Green
Aspects of Magnetic Molecularly Imprinted Polymers in Pesticide Residue
Detection. Sci. Total Environ.

[28] Bhakta S., Mishra P. (2021). Molecularly Imprinted
Polymer-Based Sensors for Cancer
Biomarker Detection. Sens. Actuators Rep.

[29] Mostafiz B., Bigdeli S. A., Banan K., Afsharara H., Hatamabadi D., Mousavi P., Hussain C. M., Keçili R., Ghorbani-Bidkorbeh F. (2021). Molecularly Imprinted Polymer-Carbon
Paste Electrode
(MIP-CPE)-Based Sensors for the Sensitive Detection of Organic and
Inorganic Environmental Pollutants: A Review. Trends Environ. Anal. Chem.

[30] BelBruno J. J. (2019). Molecularly
Imprinted Polymers. Chem. Rev.

[31] Miao W. (2008). Electrogenerated
Chemiluminescence and Its Biorelated Applications. Chem. Rev.

[32] Hu L., Xu G. (2010). Applications and Trends in Electrochemiluminescence. Chem. Soc. Rev.

[33] Parajuli S., Miao W. (2009). Sensitive Determination of Hexamethylene Triperoxide Diamine Explosives,
Using Electrogenerated Chemiluminescence Enhanced by Silver Nitrate. Anal. Chem.

[34] Glasscott M. W., Vannoy K. J., Iresh Fernando P. U.
A., Kosgei G. K., Moores L. C., Dick J. E. (2020). Electrochemical Sensors for the Detection
of Fentanyl and Its Analogs: Foundations and Recent Advances. TrAC, Trends Anal. Chem..

[35] Miao, W. Electrogenerated Chemiluminescence. Handbook of Electrochemistry. Zoski, C. G. , Ed.; Elsevier, 2007, pp. 541–590.

[36] Motchaalangaram J. A., Mahalingam P., Wallace K. J., Miao W. (2025). Electrogenerated Chemiluminescence
Coupled with Molecularly Imprinted Polymer for Sensitive and Selective
Detection of N,N-Dimethyltryptamine. Anal. Chem.

[37] Tong M., Pillai R. G., Kobryn A., Yan Z., Chan N. W. C., Jemere A. B. (2025). A Polydopamine-Based Molecularly
Imprinted Electrochemical
Sensor for Fentanyl Determination. ACS Omega.

[38] Asadi Z., Esrafili M. D., Vessally E., Asnaashariisfahani M., Yahyaei S., Khani A. (2017). A Structural Study
of Fentanyl by
DFT Calculations, NMR and IR spectroscopy. J.
Mol. Struct.

[39] Elgrishi N., Rountree K. J., McCarthy B. D., Rountree E. S., Eisenhart T. T., Dempsey J. L. (2018). A Practical Beginner’s Guide to Cyclic Voltammetry. J. Chem. Educ.

[40] Organic Electrochemistry, Hammerich, O. ; Lund, H. , Eds.; Marcel Dekker, Inc., 2001. DOI: 10.1201/9781420029659.

[41] Miao W., Choi J.-P., Bard A. J. (2002). Electrogenerated
Chemiluminescence
69: The Tris­(2,2‘-bipyridine)­ruthenium­(II), (Ru­(bpy)_3_
^2+^)/Tri-*n*-propylamine (TPrA) System Revisited--A
New Route Involving TPrA^•+^ Cation Radicals. J. Am. Chem. Soc.

[42] Mishra R. K., Goud K. Y., Li Z., Moonla C., Mohamed M. A., Tehrani F., Teymourian H., Wang J. (2020). Continuous Opioid Monitoring
along with Nerve Agents on a Wearable Microneedle Sensor Array. J. Am. Chem. Soc.

[43] Marenco A. J., Pillai R. G., Harris K. D., Chan N. W. C., Jemere A. B. (2024). Electrochemical
Determination of Fentanyl Using Carbon Nanofiber-Modified Electrodes. ACS Omega.

[44] Del
Vecchio G., Labuz D., Temp J., Seitz V., Kloner M., Negrete R., Rodriguez-Gaztelumendi A., Weber M., Machelska H., Stein C. (2019). p*K*
_a_ of Opioid Ligands as a Discriminating Factor for Side
Effects. Sci. Rep.

[45] Adenier A., Chehimi M. M., Gallardo I., Pinson J., Vilà N. (2004). Electrochemical
Oxidation of Aliphatic Amines and Their Attachment to Carbon and Metal
Surfaces. Langmuir.

[46] Kiss L., Kunsági-Máté S. (2019). Electrochemical
Oxidation of Benzaldehyde
and Hydroxybenzaldehydes in Acetonitrile on Platinum and Gassy Carbon
Electrodes. C. R. Chim.

[47] Bard, A. J. ; Faulkner, L. R. Electrochemical Methods: Fundamentals and Applications; Wiley, 2001.

[48] Ziyatdinova G., Antonova T., Davletshin R. (2023). Voltammetric Sensor Based on the
Poly­(p-aminobenzoic Acid) for the Simultaneous Quantification of Aromatic
Aldehydes as Markers of Cognac and Brandy Quality. Sensors.

[49] Ziyatdinova G., Titova M., Davletshin R. (2022). Electropolymerized
4-Aminobenzoic
Acid Based Voltammetric Sensor for the Simultaneous Determination
of Food Azo Dyes. Polymer.

[50] Thiemann C., Brett C. M. A. (2001). Electrosynthesis and Properties of Conducting Polymers
Derived from Aminobenzoic Acids and from Aminobenzoic Acids and Aniline. Synth. Met.

[51] Benyoucef A., Huerta F., Ferrahi M. I., Morallon E. (2008). Voltammetric
and In
Situ FT-IRS Study of the Electropolymerization of o-Aminobenzoic Acid
at Gold and Graphite Carbon Electrodes: Influence of pH on the Electrochemical
Behaviour of Polymer Films. J. Electroanal.
Chem.

[52] Rodrigues L. P., Ferreira D. C., Sonoda M. T., Madurro A. G. B., Abrahão O., Madurro J. M. (2014). Electropolymerization Mechanisms of Hydroxyphenylacetic
Acid Isomers. J. Mol. Struct.

[53] da
Cruz Santos C., Pimenta T. C., Thomasini R. L., Verly R. M., Franco D. L., Ferreira L. F. (2019). Electropolymerization
of Phenol and Aniline derivatives: Synthesis, Characterization and
Application as Electrochemical Transducers. J. Electroanal. Chem.

[54] Asuero A. G., Recamales A. F. (1993). A Bilogarithmic Method for the Spectrophotometric Evaluation
of Acidity Constants of Two-Step Overlapping Equilibria. Anal. Lett.

[55] National Institute of Standards and Technology, U.S. Department of Commerce. NIST Chemistry WebBook, NIST Standard Reference Database 69; National Institute of Standards and Technology, U.S. Department of Commerce. https://webbook.nist.gov/cgi/cbook.cgi?ID=C150130&Type=IR-SPEC&Index=1. Accessed 8 December 2025.

[56] Silverstein, R. M. ; Webster, F. X. ; Kiemle, D. J. ; Bryce, D. L. Spectrometric Identification of Organic Compounds; Wiley, 2015.

[57] Li M., Chen H., Xu A., Duan S., Liu Q., Zhang R., Wang S., Bai H. (2024). High-Performance Fentanyl
Molecularly Imprinted Electrochemical Sensing Platform Designed Through
Molecular Simulations. Anal. Chim. Acta.

[58] Sroysee W., Chunta S., Amatatongchai M., Lieberzeit P. A. (2019). Molecularly
Imprinted Polymers to Detect Profenofos and Carbofuran Selectively
with QCM Sensors. Phys. Med.

[59] Budd P., Makhseed S., Ghanem B., Msayib K., Tattershall C., McKeown N. (2004). Microporous Polymeric Materials. Mater. Today.

[60] Ma Y., Liu X., Guan X., Li H., Yusran Y., Xue M., Fang Q., Yan Y., Qiu S., Valtchev V. (2019). One-pot Cascade
Syntheses of Microporous and Mesoporous Pyrazine-linked Covalent Organic
Frameworks as Lewis-Acid Catalysts. Dalton Trans.

[61] Abonamah J. V., Eckenrode B. A., Moini M. (2019). On-site Detection of Fentanyl and
Its Derivatives by Field Portable Nano-Liquid Chromatography-Electron
lonization-Mass Spectrometry (nLC-EI-MS). Forensic
Chem.

[62] Joshi P., Riley P. R., Mishra R., Azizi Machekposhti S., Narayan R. (2022). Transdermal Polymeric Microneedle
Sensing Platform
for Fentanyl Detection in Biofluid. Biosensors.

[63] Dai H., Xu H., Wu X., Chi Y., Chen G. (2009). Fabrication of a New
Electrochemiluminescent Sensor for Fentanyl Citrate Based on Glassy
Carbon Microspheres and Ionic Liquid Composite Paste Electrode. Anal. Chim. Acta.

[64] Li Y., Luo L., Kong Y., Li Y., Wang Q., Wang M., Li Y., Davenport A., Li B. (2024). Recent Advances in Molecularly Imprinted
Polymer-based Electrochemical Sensors. Biosens.
Bioelectron.

[65] Chakraborty D., Chattaraj P. K. (2021). Conceptual
Density Functional Theory Based Electronic
Structure Principles. Chem. Sci.

