# Supplementary material for: Molecularly Imprinted Polymer-Based Electrogenerated Chemiluminescence Sensor for Sensitive and Selective Fentanyl Detection
Source: Anal Chem. 2026 Jan 7;98(2):1667–78. doi: 10.1021/acs.analchem.5c06407 (PMC12824990; doi:10.1021/acs.analchem.5c06407)
Supplement: Supplementary file 1 [file ac5c06407_si_001.pdf]

Supporting Information:

Molecularly Imprinted Polymer-Based Electrogenerated Chemiluminescence Sensor for Sensitive and Selective Fentanyl Detection

Arati Biswakarma and Wujian Miao*

Department of Chemistry and Biochemistry, The University of Southern Mississippi, Hattiesburg, Mississippi 39406, United States; * Corresponding author: Email: wujian.miao@usm.edu; Phone: 601-266 4716

Table of Contents

EXPERIMENTAL SECTION S-3

Reagents. S-3

Fentanyl synthesis and characterization. S-3

Scheme S1. Steps involved in one-pot synthesis of fentanyl. S-3

Figure S1. ^{1}H NMR spectra of fentanyl S-4

Figure S2. FTIR spectra of fentanyl S-4

Figure S3. ESI-MS spectra of fentanyl: (A) low-resolution MS spectra and (B) MS/MS spectra .. S-5

RESULTS AND DISCUSSION S-5

1. ECL studies on bare GCE. S-5

Figure S4. ECL intensity versus the logarithm of $[Ru(bpy)_3]^{2+}$ concentration. S-5

2. Fourier transform infrared spectroscopy (FTIR). S-5

Figure S5. ATR-FTIR spectra of (a) solid 4-ABA monomer, (b) a gold substrate on silicon wafer (Au/Si), (c) MIP film electrodeposited on an Au/Si, and (d) NIP film electrodeposited on au Au/Si. S-6

3. Elution of fentanyl from MIP films using various solvents, methods, and time periods S-6

Table S1. ECL responses of MIP films prepared with 50.0 μM fentanyl after elution using various approaches. S-6

4. DFT studies for selection of protic and aprotic polar solvents for fentanyl template elution. .. S-7

4.1 Solvation energy, dipole moments, and polarizability studies. S-7

Figure S6. Comparison of (A) solvation energy, (B) dipole moments, and (C) polarizability of fentanyl in various protic polar solvents (H_2O, MeOH, EtOH, HAc, IPA) and aprotic polar solvents (CH_3CHO, acetone, $CHCl_3$, DMF, DMSO, THF, and MeCN). S-7

4.2. Density of states (DOS) and $E_{(HOMO-LUMO)}$ gap studies. S-8

Figure S7. (A) DOS spectrum for fentanyl in the aqueous phase. (B) $E_{(HOMO-LUMO)}$ gap comparison for fentanyl in solvation phases with various protic and aprotic polar solvents. S-8

5. Comparison of limit of dection (LOD) of fentanyl. S-9

Table S2. Comparison of limit of dection (LOD) of fentanyl using various detection methods..... S-9

6. Comparison of the imprinting factor (IF) of MIP sensors... S-9

Table S3. Comparison of the imprinting factor (IF) of this fentanyl sensor against other MIP-based studies.. S-9

7. DFT studies for investigating interactions between fentanyl and its interferents and polymers in MIP systems and the selectivity of MIP toward target... S-9

Figure S8. Molecular structure of fentanyl with N and O atoms labeled.................................. S-10

7.1. Interactions of fentanyl with three units of monomers, dimers, and trimers in the gas phase, and with trimers in the aqueous phase... S-10

Figure S9. Binding energy and dipole moment of the complexes formed from fentanyl with three units of the 4-ABA monomers, dimers, and trimers in gas phase, as well as three trimers in aqueous phase. S-10

7.2. Selectivity study based on interactions of fentanyl and interferents with three trimers in gas and aqueous phases.... S-10

Figure S10. Determination of (A) chemical hardness and (B) chemical softness of complexes formed fentanyl and interferents with three units of 4-ABA trimers in gas and aqueous phases. . S-11

Figure S11. Comparison of (A) binding energy and (B) dipole moment of fentanyl and interferents with three 4-ABA trimers in gas and aqueous phases. S-12

Figure S12. The analysis of (A) Ionization potential, (B) chemical potential, and (C) electrophilicity index induced by the interactions of fentanyl and its interferents) with three units of 4-ABA trimers in gas and aqueous phases.. S-12

Figure S13. (A) DOS spectra comparing energy shifts, along with HOMO and LUMO levels, for 4-ABA trimers, fentanyl, and the complexes formed between fentanyl, interferents and three units of 4-ABA trimers in the aqueous phase. (B) HOMO-LUMO gap [$E_{(HOMO-LUMO)}$] studies for the complexes in both gas and aqueous phases. S-13

Figure S14. Diagrammatic representation of fentanyl interactions (H-bonding) with three 4-ABA trimers in aqueous phase showing: (A) HOMO and E_{HOMO}, (B) LUMO and E_{LUMO}, (C) Density of states, (D) Solvation cavities, (E) Fentanyl with trimers inside solvation cavities, and (F) Molecular electro-static potential (ESP)... S-13

References.. S-14

EXPERIMENTAL SECTION

Reagents. Disodium phosphate (Na_2HPO_4, 99.0%), triethylamine (99%), potassium ferrocyanide trihydrate ($K_4[Fe(CN)_6]•3H_2O$, 99.99+%), potassium ferricyanide ($K_3[Fe(CN)_6]$, 99+%), ferrocene methanol (97%), and 4-piperidone monohydrate hydrochloride (98%) from Sigma-Aldrich (St. Louis, MO); sodium dihydrogen phosphate monohydrate ($NaH_2PO_4•H_2O$, 99.5%) and potassium nitrate (KNO_3, 99.9%) from J.T. Bakers Chemicals (Phillipsburg, NJ); potassium chloride (KCl, ≥99.1%) from Fisher Scientific (Fair Lawn, NJ); phenylacetaldehyde (95%) from Alfa Aesar (Lancashire, UK); and tris (2,2′-bipyridyl) ruthenium (II) chloride hexahydrate ($[Ru(bpy)_3]Cl_2•6H_2O$, >98.0%) from TCI (Tokyo, Japan) were used as received. The following standard reference solutions—fentanyl (1.0 mg/mL in methanol), heroin (1.0 mg/mL in MeCN), morphine (1.0 mg/mL in MeCN), norfentanyl oxalate (1.0 mg/mL in methanol), and furanyl fentanyl (100 µg/mL in methanol)—were purchased from MilliporeSigma (Burlington, MA). The standard reference material para-methoxy-butyryl fentanyl (hydrochloride) (100 µg/mL in methanol) was acquired from Cayman Chemical (Ann Arbor, MI). To eliminate potential redox interference from methanol and MeCN, all standard reference solutions were reconstituted in 0.10 M phosphate buffer solution (PBS) at pH 7.5 after the initial solvents were slowly evaporated under a nitrogen stream.

Fentanyl synthesis and characterization.

Scheme S1. Steps involved in one-pot synthesis of fentanyl.

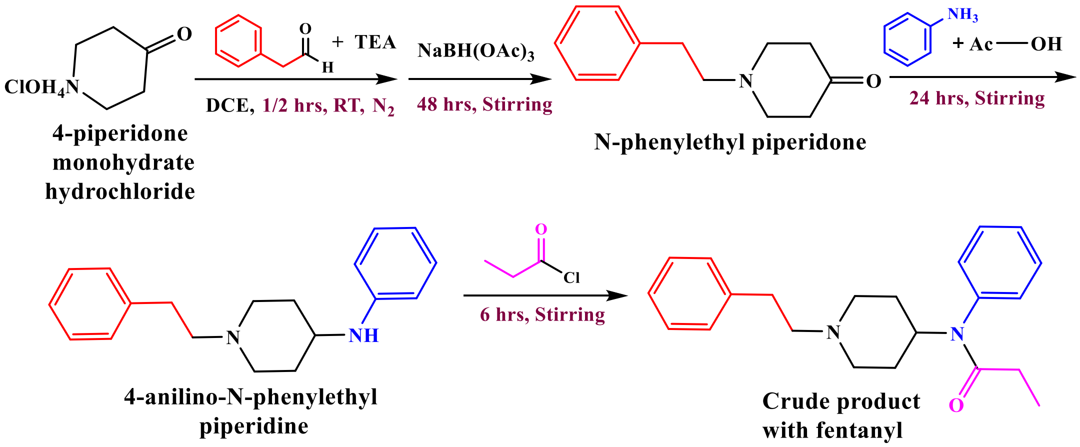

Fentanyl is highly restricted and regulated due to its potency and potential for abuse, limiting its commercial availability for laboratory research. In this study, fentanyl was synthesized using a modified one-pot approach based on Asadi et al.[1] Specifically, 4-piperidone monohydrochloride (500 mg, 3.26 mmol) was dissolved in dichloroethane (14.65 mL) at room temperature, followed by the addition of triethylamine (907.4 µL, 6.51 mmol) and phenylacetaldehyde (363.8 µL, 3.26 mmol). The mixture was stirred for 30 min under a nitrogen atmosphere before adding sodium triacetoxyborohydride (1.45 g, 6.84 mmol). The reaction proceeded with stirring under nitrogen for 48 h. Subsequently, aniline (296.9 µL, 3.26 mmol), glacial acetic acid (375.9 µL, 6.51 mmol), and a second portion of sodium triacetoxyborohydride (1.45 g, 6.84 mmol) were added, and the reaction continued for an additional 24 h under the same conditions. Finally, propionyl chloride

(1.70 mL, 19.53 mmol) was added dropwise, and the crude fentanyl was obtained after 6 h of further reaction.

The reaction mixture was diluted with dichloromethane, washed subsequentially with 4% aqueous NaOH and water, and extracted with 2.0 M HCl. The aqueous layer was separated, and the organic layers were combined, dried over anhydrous sodium sulfate, and evaporated under reduced pressure to yield crude fentanyl hydrochloride. Recrystallization from acetone afforded fentanyl hydrochloride as a white powder, which was treated with 20% aqueous NaOH to liberate the free base, followed by recrystallization from petroleum ether (b.p. 60–80 °C) to yield pure fentanyl with an overall 11.5% yield.

Figure S1 shows the ^{1}H NMR spectra of fentanyl, with the following chemical shifts corresponding to specific protons: ^{1}H NMR (400 MHz, $CDCl_3$) δ 7.32 – 7.00 (m, 10H, aromatic), 4.65 – 4.59 (m, 2H), 2.69 – 2.65 (m, 2H), 2.50 – 2.46 (m, 2H), 2.14 – 2.08 (t, J = 12 Hz, 2H), 1.89 – 1.83 (q, J = 8 Hz, 2H), 1.76 – 1.73 (m, 2H), 1.43 -1.34 (m, 2H), 0.96 - 0.93 (t, J = 6 Hz, 3H).

Figure S2 shows the FTIR spectra of fentanyl, with the following characteristic stretches: C=O stretch at 1645.7 cm^{-1}, sp^2 C-H stretches at 3000-3100, sp^3 C-H stretches at 2850-2960 and 2933.8 cm^{-1}, and C-H bending from the monosubstituted phenyl rings at 702.8 cm^{-1}.

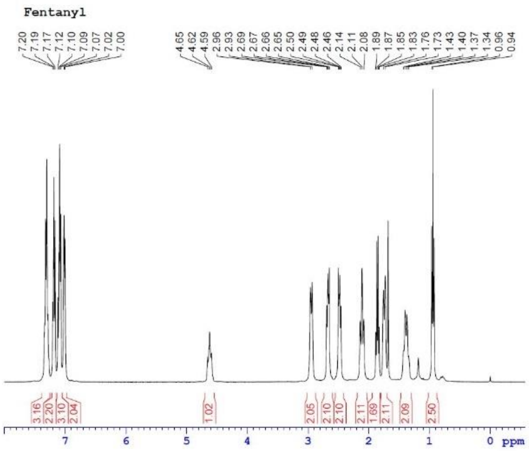

Figure S1. ^{1}H NMR spectra of fentanyl, acquired using a Bruker Ultra Shield Plus 400 MHz spectrometer.

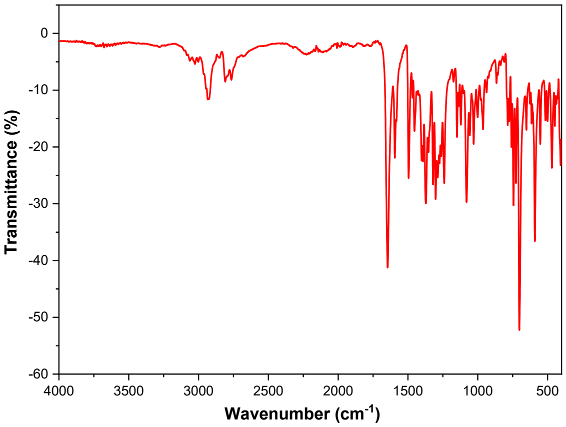

Figure S2. FTIR spectra of fentanyl, acquired using a Nicolet Summit FTIR spectrometer from Thermo Fisher Scientific.

The ESI-MS spectra of fentanyl, presented in Figure S3, along with the NMR and FTIR data, confirm the molecular structure of the synthesized compound. The low-resolution MS spectra (Figure S3(A)) shows the protonated molecular ion [$M+H^+$] at m/z 337.20, closely matching the calculated value of 337.23 for $C_{22}H_{28}N_2O{\bullet}H^+$. The MS/MS spectra (Figure S3(B)) displays

characteristic fragment ions at m/z 105, 188, and 281, consistent with fentanyl's fragmentation pattern, as reported.[2]

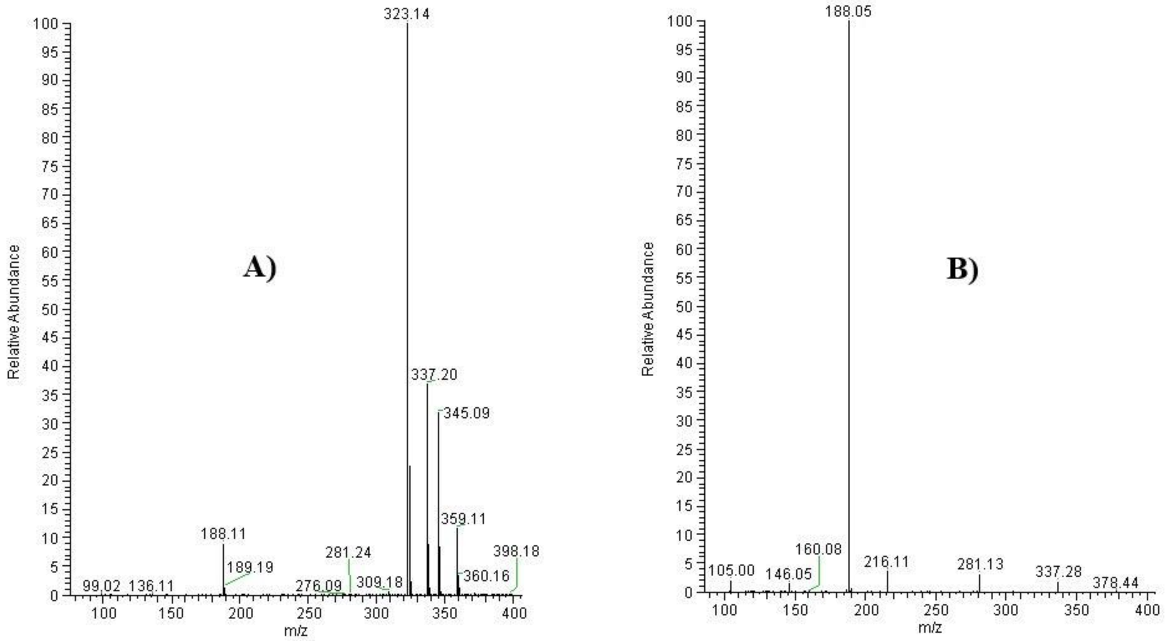

Figure S3. ESI-MS spectra of fentanyl: (A) low-resolution MS spectra and (B) MS/MS spectra, acquired using a Thermo Finnigan LXQ linear ion trap spectrometer.

RESULTS AND DISCUSSION

1. ECL studies on bare GCE.

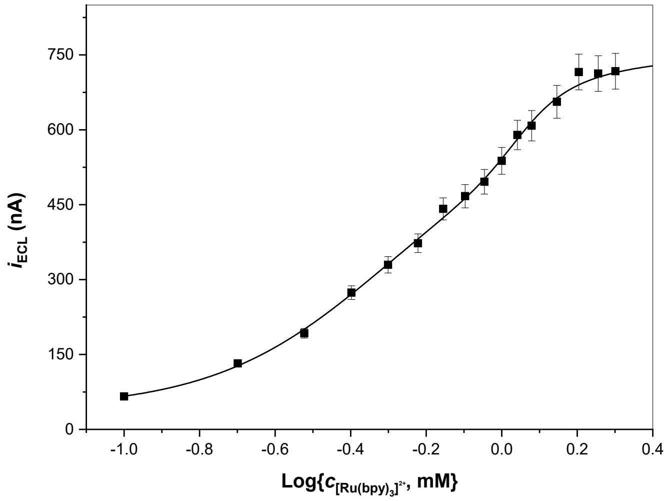

Figure S4. ECL intensity versus the logarithm of $[Ru(bpy)_3]^{2+}$ concentration with 50 µM fentanyl in 0.10 M PBS (pH 7.5), measured with cyclic voltammetry (0 to 1.60 V vs. Ag/AgCl) at a glassy carbon electrode and a scan rate of 50 mV/s.

2. Fourier transform infrared spectroscopy (FTIR).

FTIR measurements were performed using a Thermo Fisher Everest™ Nicolet™ Summit diamond crystal ATR-FTIR spectrometer, scanning from 400 to 4000 cm^{-1} with 128 scans. The MIP film,

(4-ABA)$_n$⊂Fent, was electrodeposited onto a gold-coated silicon wafer from a solution containing 4.0 mM 4-ABA, 1.0 mM fentanyl, and 0.10 M PBS (pH 7.5). Electrodeposition was carried out by cycling the potential from -0.20 to1.0 V vs. Ag/AgCl at a scan rate of 50 mV for 20 cycles. A similar procedure was used to prepare the NIP film on gold-coated silicon in the absence of fentanyl.

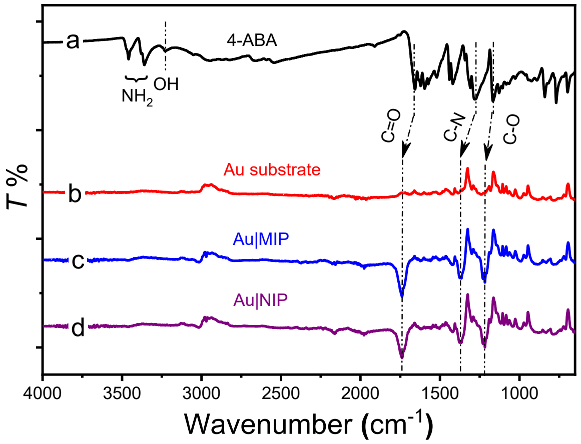

Figure S5. ATR-FTIR spectra of (a) solid 4-ABA monomer, (b) a gold substrate on silicon wafer (Au/Si), (c) MIP film electrodeposited on an Au/Si, and (d) NIP film electrodeposited on au Au/Si.

3. Elution of fentanyl from MIP films using various solvents, methods, and time periods.

Table S1. ECL responses of MIP films prepared with 50.0 µM fentanyl after elution using various approaches.

Solvents	Methods	Time (min)	ECL Intensity (nA)
MeOH	Dipping	2	60.0
		4	60.0
		10	25.0
		30	25.0
		45	25.0
	Swirling	3	24.7
		4	22.5
		5	15.5
	Ultrasonication	3	31.2
	Magnetic Stirring	5	13.4
0.10 M PBS buffer, pH 7.5	Ultrasonication	3	20.0
MeOH:Pure HAc (50:50, v/v)	Magnetic Stirring	3	15.9
		4	13.9
		5	12.13
MeOH:2.0 M H_2SO_4 (1:1, v/v)	Dipping	0.5	16.0
		4	14.0
		6	13.8
		8	12.0
MeOH: 2.0 M H_2SO_4 (9:1, v/v)	Dipping	1.5	16.5

4. DFT studies for selection of protic and aprotic polar solvents for fentanyl template elution.

Density functional theory (DFT) calculations were performed to optimize the fentanyl structure in the gas phase and in various protic and aprotic polar solvents, aiding the selection of suitable solvents for eluting fentanyl from GCE|MIP⊏Fent electrodes. The calculation employed Becke's three-parameter exchange functional with the Lee–Yang–Parr correlation (B3LYP) functional and the 6-311++G(d,p) basis set.[1, 3, 4] Solvation energies were calculated using the universal solvation model (SMD)[5, 6] with the equation shown in Eq. S1.[7] The HOMO-LUMO energy gap ($E_{(HOMO-LUMO)}$) gap was determined from the density of states (DOS), and DOS spectra were plotted using GaussSum 3.0.2 software.[8]

4.1 Solvation energy, dipole moments, and polarizability studies.

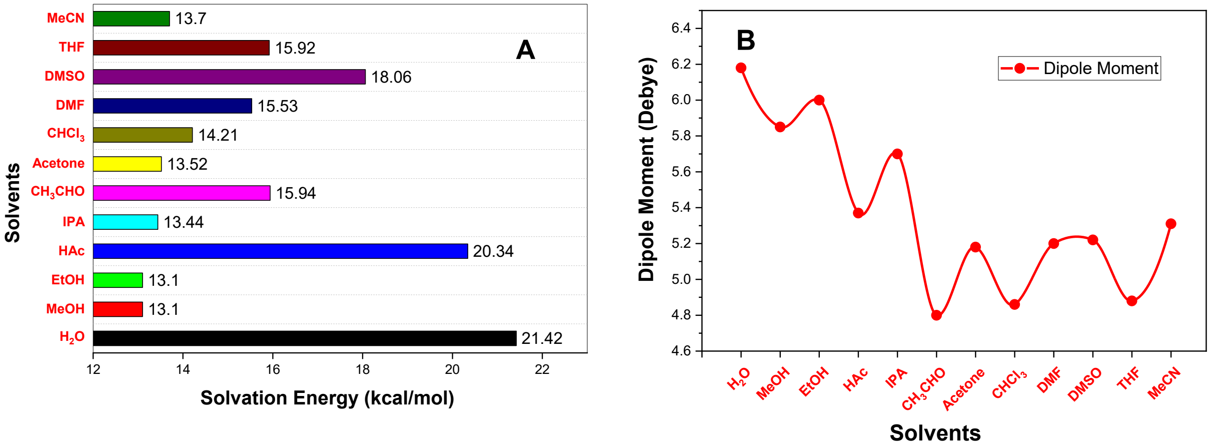

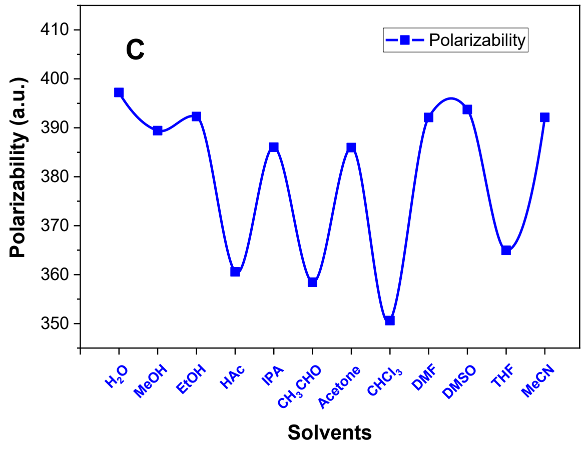

Figure S6. Comparison of (A) solvation energy, (B) dipole moments, and (C) polarizability of fentanyl in various protic polar solvents (H_2O, MeOH, EtOH, HAc, IPA) and aprotic polar solvents (CH_3CHO, acetone, $CHCl_3$, DMF, DMSO, THF, and MeCN).

$$\Delta G_{solvation} = E_{(\text{Optimized in solvent phase})} - E_{(\text{Optimized in gas phase})} \quad \text{(S1)}$$

Where $\Delta G_{solvation}$ is the solvation free energy, $E_{(\text{Optimized in solvent phase})}$ is the total energy of fentanyl in the solvent, and $E_{(\text{Optimized in gas phase})}$ is the total energy in the gas phase. For fentanyl, the optimized gas-phase energy was -1040.2621 Hartree (1 Hartree = 2625.5 KJ/mol = 627.509 kcal/mol = 27.211 eV).

In Figure S6A, methanol and ethanol exhibit the lowest positive solvation energies among all calculated protic (water, methanol, ethanol, acetic acid, isopropanol) and aprotic (acetaldehyde, acetone, chloroform, DMF, DMSO, THF, acetonitrile) polar solvents, indicating strong stabilization and high solubility of fentanyl in these solvents. This facilitates effective elution from the GCE|MIP⊏Fent electrodes. Compared to ethanol, methanol has a smaller dipole moment and lower polarizability, as shown in Figures S6B and S6C, respectively, making it an effective solvent for fentanyl removal while minimizing disruption to the 4-ABA polymer matrix.

4.2. Density of states (DOS) and $E_{(HOMO\text{-}LUMO)}$ gap studies.

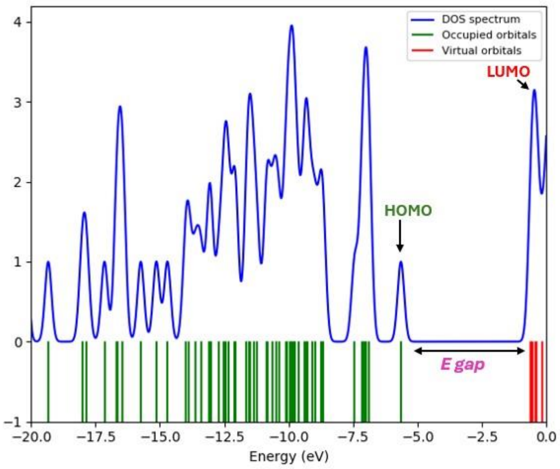

(A)

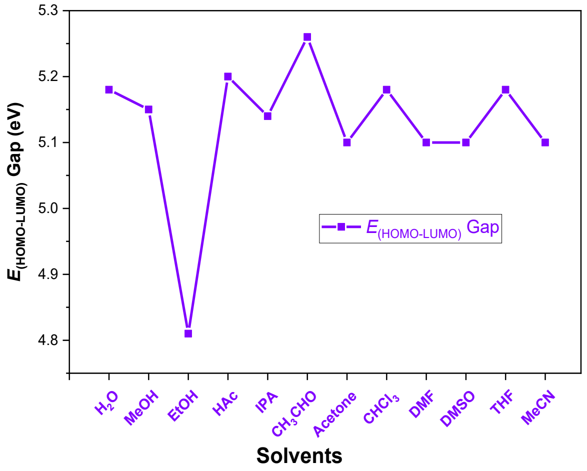

(B)

Figure S7. (A) DOS spectrum for fentanyl in the aqueous phase. (B) $E_{(HOMO\text{-}LUMO)}$ gap comparison for fentanyl in solvation phases with various protic and aprotic polar solvents.

The HOMO-LUMO gaps ($E_{(HOMO\text{-}LUMO)}$) were derived from DOS spectra. Figure S7A presents the DOS spectrum for fentanyl in water. As shown in Figure S7B, methanol exhibits a higher HOMO-LUMO gap than ethanol, indicating lower chemical reactivity and greater stability of fentanyl in methanol. Combining the lowest positive solvation free energies (Figure S6A) with the higher HOMO-LUMO energy gap (Figure S7B), methanol emerges as the most suitable solvent for fentanyl elution, effectively solubilizing the template while preserving MIP cavity integrity. Although acetic acid (HAc) alone may not be used as an efficient elution solvent due to higher solvation energy, its addition to methanol enhances elution by creating an acidic medium that disrupts the hydrogen bonding between fentanyl (template) and the 4-ABA matrix through competitive binding.[9, 10] These DFT and DOS results support the experimental selection of methanol-based solvents, confirming effective template removal without compromising the MIP structure.

5. Comparison of limit of dection (LOD) of fentanyl.

Table S2. Comparison of limit of dection (LOD) of fentanyl using various detection methods.

Methods	LOD (μM)	References
1. Electrochemical		
Graphene modified microneedle - SWV	27.8	Joshi et al.[11]
Screen printed-MWCNTs - SWV	10	Barfidokht et al.[12]
Aptamer based - SWV	0.01	Canoura et al.[13]
SPCE with MOF - CV and DPV	0.3	Naghian et al.[14]
2. Visual calorimetry	2.08	Lin et al.[15]
3. Surface enhanced Raman spectroscopy (SERS)	0.14	Su et al.[16]
4. Lateral flow chromatographic immunoassay	0.59	Park et al.[17]
5. Nano-LC-EI-MS	0.004	Abonamah et al.[18]
6. ECL with glassy carbon paster electrode	0.0085	Dai et al[19]
7. MIP-ECL sensor	~1	This work

6. Comparison of the imprinting factor (IF) of MIP sensors.

Table S3. Comparison of the imprinting factor (IF) of this fentanyl sensor against other MIP-based studies.

Material Type	Technique	IF	Target Analyte	Reference
MIP -AuNPs/RGO	DPV	3.09	Nitrofurazone	Zhou et al.[20]
MIP-Nano sensor	SPR	4.14	Adenosine	Kurt et al.[21]
MIP-ppy /TiO_2	DPV	2.30	Porcine-DNA	Nawaz et al.[22]
MIP-MagneticNPs/QDs	FL	4.12	Lysozyme	Zhang et al.[23]
MIP-CoNi/MOF-RGO-GCE	DPV	6.53	Hippuric acid	Gao et al.[24]
MIP-ppy GCE-BP-PEDOT:PSS	LSV	3.86	Norfloxacin	Li et al.[25]
MIP-4-ABA/Nafion-$[Ru(bpy)_3]^{2+}$/GCE	ECL	6.60	DMT	Motchaalangaram[9]
MIP-4-ABA/GCE, $[Ru(bpy)_3]^{2+}$	ECL	9.92	Fentanyl	This study

7. DFT studies for investigating interactions between fentanyl and its interferents and polymers in MIP systems and the selectivity of MIP toward target.

The optimization of fentanyl with 4-ABA monomer and the corresponding polymer structures was performed in both gas and aqueous (H_2O) phases using DFT. The B3LYP functional with 6-311++G(d,p)[26, 27] basis set for single and small molecules, whereas the 6-31g(d)[7, 28] basis set was used for modeling interactions between fentanyl and polymer fragments in both gas and aqueous phases. All calculations incorporated Grimme's D3 dispersion correction method.[29] The interaction between fentanyl and the polymer matrix primarily governed by the hydrogen bond

accepting capacity of fentanyl. Three hydrogen bonds were identified, two stronger ones involving O_9 and N_{39}, and one weaker involving N_8 (Figure S8),[30] with average bond lengths of approximately 2 Å. In contrast, the optimized structure of fentanyl interactions with three trimers exhibited only two hydrogen bonds of similar length to the previous model, while the N_8-associated bond length exceeded 3 Å, indicating a considerable weaker interaction than typical hydrogen bonding.

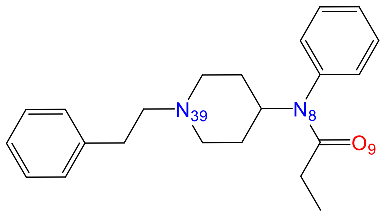

Figure S8. Molecular structure of fentanyl with N and O atoms labeled.

The binding energy of the template (fentanyl or the interferent), ΔE_b, was calculated using Eq. S2:[9]

$$\Delta E_b = E_{\text{Template}} + n \bullet E_{\text{Trimer}} - E_{\text{Template/Trimer matrix}} \qquad \text{(S2)}$$

where E_{Template} is the energy of the optimized geometry of the template (i.e., fentanyl or the interferent), n (= 3) is the number of the 4-ABA trimer involved in the calculation, E_{Trimer} is the energy of the optimized geometry of the trimer, and $E_{\text{Template/Trimer matrix}}$ is the energy of optimized geometry of the template interacting with the trimer matrix.

7.1. Interactions of fentanyl with three units of monomers, dimers, and trimers in the gas phase, and with trimers in the aqueous phase.

DFT calculations reveal that the complex formed between fentanyl and three 4-ABA trimers [3×(4-ABA)$_3$] in water exhibits the most negative binding energy and the highest dipole moment among all complexes examined in both gas and aqueous phases (Figure S9). Therefore, subsequent analyses focus on the interactions of the three trimers with fentanyl in gas and aqueous phases to better elucidate the MIP's selectivity toward the target fentanyl molecule and potential interferents.

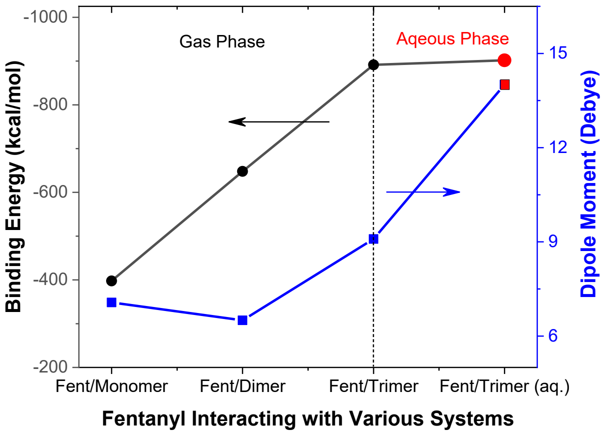

Figure S9. Binding energy and dipole moment of the complexes formed from fentanyl with three units of the 4-ABA monomers, dimers, and trimers in gas phase, as well as three trimers in aqueous phase.

7.2. Selectivity study based on interactions of fentanyl and interferents with three trimers in gas and aqueous phases.

Quantum chemical descriptors such as electron affinity (A), ionization potential or energy (IP), chemical potential (μ), chemical hardness (η) and softness (S), electrophilicity index (ω), and HOMO-LUMO energy gap ($E_{(\text{HOMO-LUMO})}$ gap or E gap), provide insights into molecular reactivity and stability. These parameters are particularly

useful for analyzing the interactions between fentanyl, its interferents, and three 4-ABA trimers in both gas and aqueous phases, enabling an assessment of sensor selectivity.

The standard derivations for these global reactivity indices, based on frontier molecular orbital theory within the framework of` conceptual DFT, are as follows:[31, 32]

$$\text{Electron Affinity } (A) = -E_{\text{LUMO}} \quad \text{(S3)}$$

$$\text{Ionization Potential } (IP) = -E_{\text{HOMO}} \quad \text{(S4)}$$

$$\text{Chemical Potential } (\mu) = -\frac{(E_{\text{HOMO}} + E_{\text{LUMO}})}{2} \quad \text{(S5)}$$

$$\text{Chemical Hardness } (\eta) = \frac{(E_{\text{LUMO}} - E_{\text{HOMO}})}{2} \quad \text{(S6)}$$

$$\text{Chemical Softness } (S) = \frac{1}{\eta} \quad \text{(S7)}$$

$$\text{Electrophilicity Index } (\omega) = \frac{\mu^2}{2\eta} \quad \text{(S8)}$$

$$E_{\text{(HOMO-LUMO)}} \text{ gap } (E \text{ gap}) = E_{\text{LUMO}} - E_{\text{HOMO}} \quad \text{(S9)}$$

(A) Chemical hardness and softness.

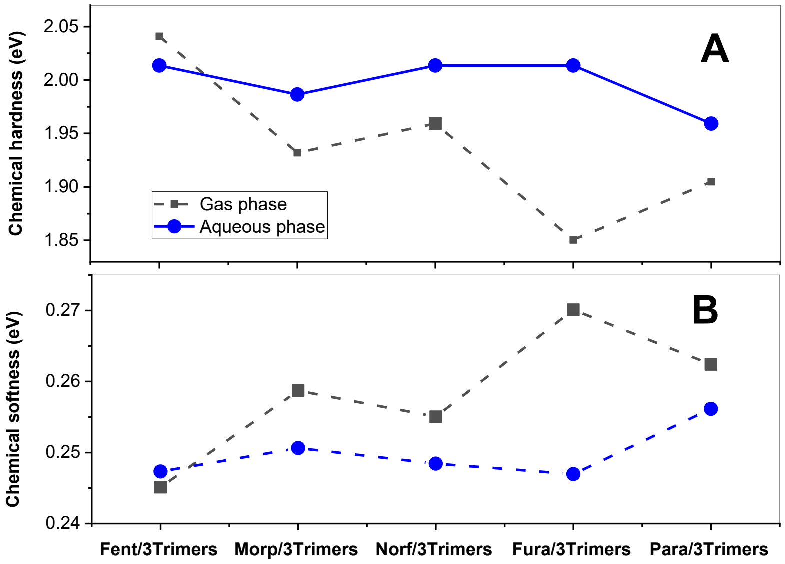

Figure S10. Determination of (A) chemical hardness and (B) chemical softness of complexes formed fentanyl and interferents with three units of 4-ABA trimers in gas and aqueous phases.

(B) Binding energy and dipole moment.

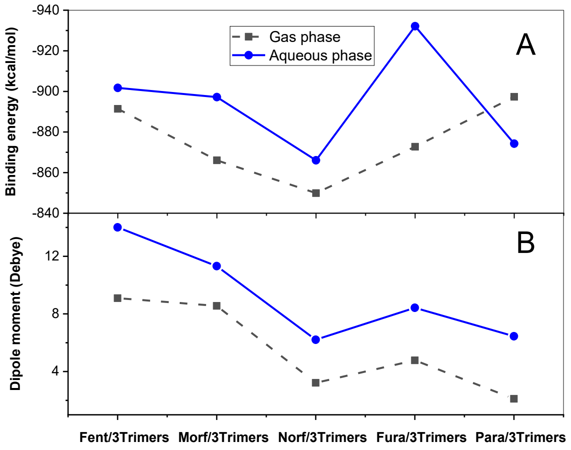

Figure S11. Comparison of (A) binding energy and (B) dipole moment of fentanyl and interferents with three 4-ABA trimers in gas and aqueous phases.

(C) Ionization potential, chemical potential, and electrophilicity.

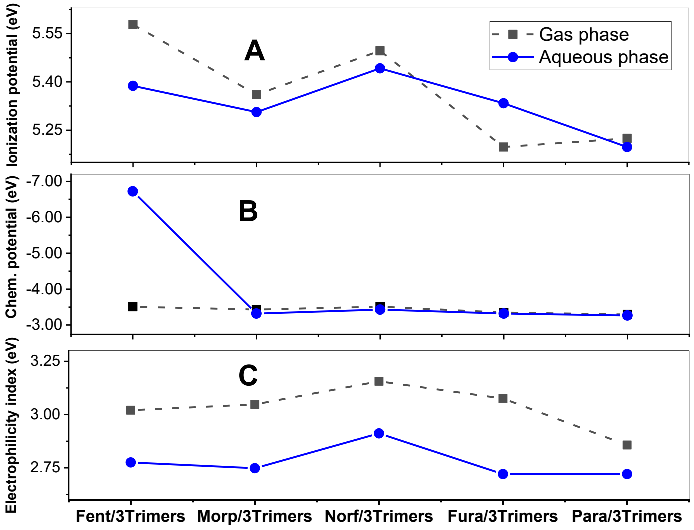

Figure S12. The analysis of (A) Ionization potential, (B) chemical potential, and (C) electrophilicity index induced by the interactions of fentanyl and its interferents) with three units of 4-ABA trimers in gas and aqueous phases.

(D) Density of states and E(HOMO-LUMO) gap studies.

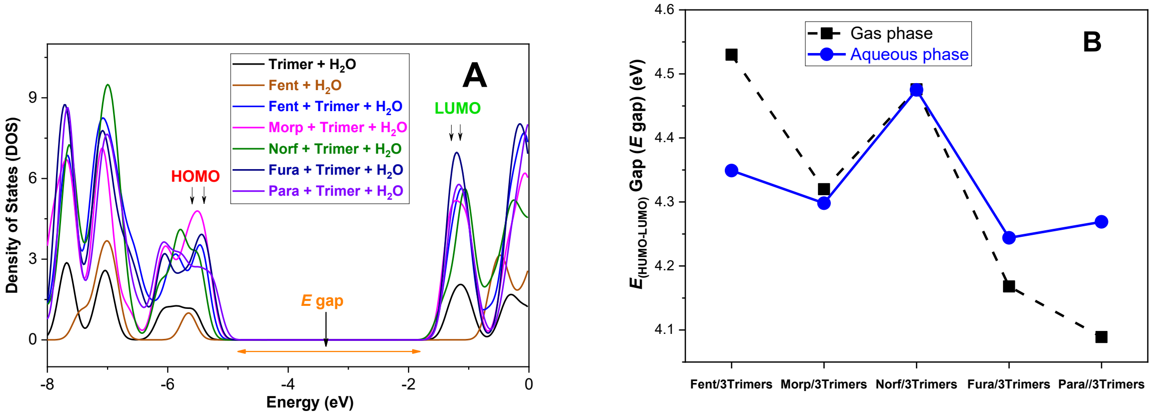

Figure S13. (A) DOS spectra comparing energy shifts, along with HOMO and LUMO levels, for 4-ABA trimers, fentanyl, and the complexes formed between fentanyl, interferents and three units of 4-ABA trimers in the aqueous phase. (B) HOMO-LUMO gap [$E_{(HOMO-LUMO)}$] studies for the complexes in both gas and aqueous phases.

A diagrammatic representation of fentanyl interactions (H-bonding) with three 4-ABA trimers in aqueous phase is shown in Figure S14.

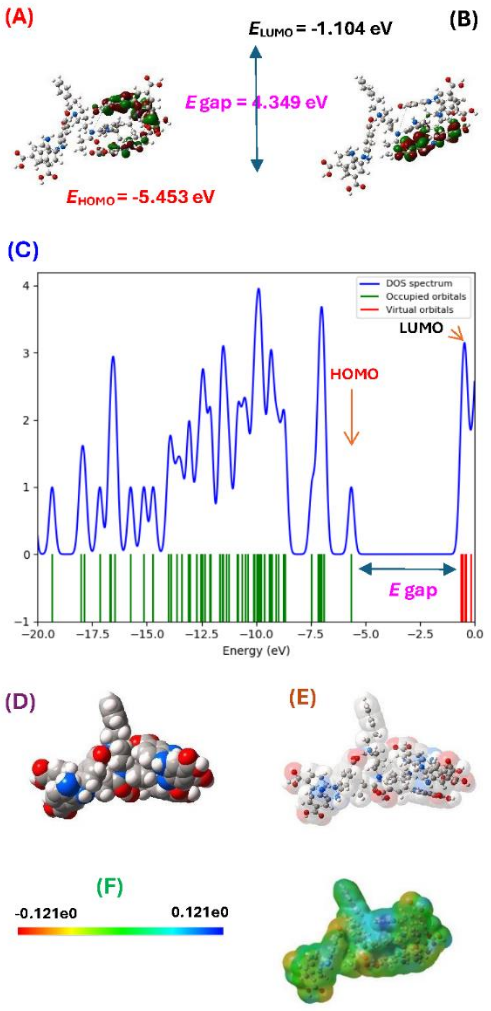

Figure S14. Diagrammatic representation of fentanyl interactions (H-bonding) with three 4-ABA trimers in aqueous phase showing:
(A) HOMO and E_{HOMO}
(B) LUMO and E_{LUMO}
(C) Density of states
(D) Solvation cavities
(E) Fentanyl with trimers inside solvation cavities.
(F) Molecular electro-static potential (ESP).

References:

(1) Asadi, Z.; Esrafili, M. D.; Vessally, E.; Asnaashariisfahani, M.; Yahyaei, S.; Khani, A. A Structural Study of Fentanyl by DFT Calculations, NMR and IR Spectroscopy. *J. Mol. Struct.* **2017**, *1128*, 552-562. DOI: 10.1016/j.molstruc.2016.09.027.

(2) W. De Silva, I.; Couch, A. N.; Verbeck, G. F. Paper Spray Mass Spectrometry Utilized with a Synthetic Microporous Polyolefin Silica Matrix Substrate in the Rapid Detection and Identification of More than 190 Synthetic Fentanyl Analogs. *J. Am. Soc. Mass Spectrom.* **2021**, *32* (2), 420-428. DOI: 10.1021/jasms.0c00250.

(3) Lau, J. K.-C.; Romanov, V.; Lukow, S.; Hopkinson, A. C.; Verkerk, U. H. Collision-Induced Dissociation of Protonated Fentanyl: A DFT Study. *Comput. Theor. Chem.* **2021**, *1196*, 113117. DOI: 10.1016/j.comptc.2020.113117.

(4) Bin, S.; Yu, J.; Tang, T.; Yuan, L.; Tang, Y. Calculation of the UV Spectrum and Electrophilic Reactive Sites of Fentanyl Molecule Based on the Density Functional Theory. *Russ. J. Phys. Chem. A* **2020**, *94* (12), 2586-2593. DOI: 10.1134/S0036024420120055.

(5) Liu, S.-C.; Zhu, X.-R.; Liu, D.-Y.; Fang, D.-C. DFT Calculations in Solution Systems: Solvation Energy, Dispersion Energy and Entropy. *Phys. Chem. Chem. Phys.* **2023**, *25* (2), 913-931, 10.1039/D2CP04720A. DOI: 10.1039/D2CP04720A.

(6) Ahmed, L.; Omer, R. The Role of the Various Solvent Polarities on Piperine Reactivity and Stability. *J. Phys. Chem. Funct. Mater.* **2021**, *4* (2), 10-16. DOI: 10.54565/jphcfum.990410 (acccessed 2021).

(7) Chittratan, P.; Phromyothin, D.; Monvisade, P. DFT-Based Computational Investigation on Functional Monomer and Solvent Selection of Molecularly Imprinted Polymers for Recognition of Chlorpyrifos Organophosphate Insecticide. *Suranaree J. Sci. Technol* **2020**, *27* (030003), 1-030003.

(8) O'Boyle, N. M.; Tenderholt, A. L.; Langner., K. M. A Library for Package-Independent Computational Chemistry Algorithms. *J. Comp. Chem.* **2008**, *29*, 839-845. DOI: 10.1002/jcc.20823.

(9) Motchaalangaram, J. A.; Mahalingam, P.; Wallace, K. J.; Miao, W. Electrogenerated Chemiluminescence Coupled with Molecularly Imprinted Polymer for Sensitive and Selective Detection of *N,N*-Dimethyltryptamine. *Anal. Chem.* **2025**, *97* (11), 6163-6174. DOI: 10.1021/acs.analchem.4c06886.

(10) Sroysee, W.; Chunta, S.; Amatatongchai, M.; Lieberzeit, P. A. Molecularly Imprinted Polymers to Detect Profenofos and Carbofuran Selectively with QCM Sensors. *Phys. Med.* **2019**, *7*, 100016. DOI: 10.1016/j.phmed.2019.100016.

(11) Joshi, P.; Riley, P. R.; Mishra, R.; Azizi Machekposhti, S.; Narayan, R. Transdermal Polymeric Microneedle Sensing Platform for Fentanyl Detection in Biofluid. In *Biosensors*, 2022; Vol. 12.

(12) Barfidokht, A.; Mishra, R. K.; Seenivasan, R.; Liu, S.; Hubble, L. J.; Wang, J.; Hall, D. A. Wearable Electrochemical Glove-Based Sensor for Rapid and On-Site Detection of Fentanyl. *Sens. Actuators, B* **2019**, *296*, 126422. DOI: 10.1016/j.snb.2019.04.053.

(13) Canoura, J.; Liu, Y.; Alkhamis, O.; Xiao, Y. Aptamer-Based Fentanyl Detection in Biological Fluids. *Anal. Chem.* **2023**, *95* (49), 18258-18267. DOI: 10.1021/acs.analchem.3c04104.

(14) Naghian, E.; Marzi Khosrowshahi, E.; Sohouli, E.; Ahmadi, F.; Rahimi-Nasrabadi, M.; Safarifard, V. A New Electrochemical Sensor for the Detection of Fentanyl Lethal Drug by a Screen-Printed Carbon Electrode Modified with the Open-Ended Channels of Zn(ii)-MOF. *New J. Chem.* **2020**, *44* (22), 9271-9277, 10.1039/D0NJ01322F. DOI: 10.1039/D0NJ01322F.

(15) Lin, Y.; Sun, J.; Tang, M.; Zhang, G.; Yu, L.; Zhao, X.; Ai, R.; Yu, H.; Shao, B.; He, Y. Synergistic Recognition-Triggered Charge Transfer Enables Rapid Visual Colorimetric Detection of Fentanyl. *Anal. Chem.* **2021**, *93* (16), 6544-6550. DOI: 10.1021/acs.analchem.1c00723.

(16) Su, X.; Liu, X.; Xie, Y.; Chen, M.; Zhong, H.; Li, M. Quantitative Label-Free SERS Detection of Trace Fentanyl in Biofluids with a Freestanding Hydrophobic Plasmonic Paper Biosensor. *Anal. Chem.* **2023**, *95* (7), 3821-3829. DOI: 10.1021/acs.analchem.2c05211.

(17) Park, J. N.; Sherman, S. G.; Sigmund, V.; Breaud, A.; Martin, K.; Clarke, W. A. Validation of a Lateral Flow Chromatographic Immunoassay for the Detection of Fentanyl in Drug Samples. *Drug Alcohol Depend.* **2022**, *240*, 109610. DOI: 10.1016/j.drugalcdep.2022.109610.

(18) Abonamah, J. V.; Eckenrode, B. A.; Moini, M. On-site Detection of Fentanyl and Its Derivatives by Field Portable Nano-Liquid Chromatography-Electron lonization-Mass Spectrometry (nLC-EI-MS). *Forensic Chem.* **2019**, *16*, 100180. DOI: 10.1016/j.forc.2019.100180.

(19) Dai, H.; Xu, H.; Wu, X.; Chi, Y.; Chen, G. Fabrication of a New Electrochemiluminescent Sensor for Fentanyl Citrate Based on Glassy Carbon Microspheres and Ionic Liquid Composite Paste Electrode. *Anal. Chim. Acta* **2009**, *647* (1), 60-65. DOI: 10.1016/j.aca.2009.05.032.

(20) Zhou, B. B.; Sheng, X. X.; Xie, H.; Zhou, S. S.; Huang, L. J.; Zhang, Z. K.; Zhu, Y. B.; Zhong, M. Molecularly Imprinted Electrochemistry Sensor Based on (AuNPs) / (RGO) Modification for Highly Sensitive and Selective Detection of Nitrofurazone. *Food Anal. Methods* **2023**, *16* (4), 709-720. DOI: 10.1007/s12161-023-02447-y.

(21) Kurt, Z. T.; Çimen, D.; Denizli, A.; Bereli, N. Development of Optical-Based Molecularly Imprinted Nanosensors for Adenosine Detection. *ACS Omega* **2023**, *8* (21), 18839-18850. DOI: 10.1021/acsomega.3c01028.

(22) Nawaz, N.; Abu Bakar, N. K.; Basirun, W. J.; Shalauddin, M.; Karman, S. B.; Ibrahim, S. B.; Mahmud, H. N. M. E. Exploration of Molecularly Imprinted Polymer (MIP) Nanohybrid Films as DNA Sensors for the Detection of Porcine. *Chem. Pap.* **2023**, *77* (2), 987-1003. DOI: 10.1007/s11696-022-02530-4.

(23) Zhang, X.; Tang, B.; Li, Y.; Liu, C.; Jiao, P.; Wei, Y. Molecularly Imprinted Magnetic Fluorescent Nanocomposite-Based Sensor for Selective Detection of Lysozyme. In *Nanomater.*, 2021; Vol. 11.

(24) Gao, H.; Chai, J.; Jin, C.; Tian, M. Molecularly Imprinted Electrochemical Sensor Based on CoNi-MOF/RGO Nanocomposites for Sensitive Detection of the Hippuric Acid. *Anal. Chim. Acta* **2024**, *1296*, 342307. DOI: 10.1016/j.aca.2024.342307.

(25) Li, G.; Wu, J.; Qi, X.; Wan, X.; Liu, Y.; Chen, Y.; Xu, L. Molecularly Imprinted Polypyrrole Film-Coated Poly(3,4-ethylenedioxythiophene):Polystyrene Sulfonate-Functionalized Black Phosphorene for the Selective and Robust Detection of Norfloxacin. *Mater. Today Chem.* **2022**, *26*, 101043. DOI: 10.1016/j.mtchem.2022.101043.

(26) Ebadi, S.; Ghanbari, K.; Zahedi-Tabrizi, M. Development of an Electrochemical Sensor Based on Ni-Bio-MOF and a Molecular Imprinted Polymer for Determination of Diclofenac: Electrochemical and DFT Investigations. *RSC Adv.* **2025**, *15* (21), 16983-16998, 10.1039/D5RA00194C. DOI: 10.1039/D5RA00194C.

(27) Umar, Y. Analysis of the Structures, Electronic, and Spectroscopic Properties of Piperidine-Based Analgesic Drugs Carfentanil and Acetylfentanyl. *Arabian J. Sci. Eng.* **2022**, *47* (1), 511-522. DOI: 10.1007/s13369-021-05791-5.

(28) Fahim, A. M.; Magd, E. E. A.-E. Enhancement of Molecular Imprinted Polymer as Organic Fillers on Bagasse Cellulose Fibers with Biological Evaluation and Computational Calculations. *J. Mol. Struct.* **2021**, *1241*, 130660. DOI: DOI: 10.1016/j.molstruc.2021.130660.

(29) Gunawan, U.; Ibrahim, S.; Luqman Ivansyah, A.; Damayanti, S. Insights Into the Selective Imprinted Polymer of Voriconazole from Host-Guest Interaction Perspective. *J. Mol. Liq.* **2023**, *383*, 122130. DOI: DOI: 10.1016/j.molliq.2023.122130.

(30) Faulkner, C.; Santos-Carballal, D.; Plant, D. F.; de Leeuw, N. H. Atomistic Molecular Dynamics Simulations of Propofol and Fentanyl in Phosphatidylcholine Lipid Bilayers. *ACS Omega* **2020**, *5* (24), 14340-14353. DOI: DOI: 10.1021/acsomega.0c00813.

(31) Pal, R.; Chattaraj, P. K. Chemical Reactivity From a Conceptual Density Functional Theory Perspective. *J. Indian Chem. Soc.* **2021**, *98* (1), 100008. DOI: DOI: 10.1016/j.jics.2021.100008.

(32) Khanlari, M.; Daraei, B.; Torkian, L.; Shekarchi, M.; Manafi, M. R. Application of the Oxycodone Templated Molecular Imprinted Polymer in Adsorption of the Drug From Human Blood Plasma as the Real Biological Environment; A Joint Experimental and Density Functional Theory Study. *Front. Chem.* **2023**, *Volume 10 - 2022*, Original Research. DOI: 10.3389/fchem.2022.1045552.
